# VPS33B interacts with NESG1 to modulate EGFR/PI3K/AKT/c-Myc/P53/miR-133a-3p signaling and induce 5-fluorouracil sensitivity in nasopharyngeal carcinoma

**DOI:** 10.1038/s41419-019-1457-9

**Published:** 2019-04-03

**Authors:** Zixi Liang, Zhen Liu, Chao Cheng, Hao Wang, Xiaojie Deng, Jiahao Liu, Chen Liu, Yonghao Li, Weiyi Fang

**Affiliations:** 1 Cancer Center, Integrated Hospital of Traditional Chinese Medicine, Southern Medical University, 510310 Guangzhou, Guangdong P. R. China; 2 Cancer Center, School of Basic Medical Science, Southern Medical University, 510310 Guangzhou, Guangdong P. R. China; 3 Key Laboratory of Protein Modification and Degradation, School of Basic Medical Sciences, Affiliated Cancer Hospital and Institute of Guangzhou Medical University, 511436 Guangzhou, P. R. China; 4 Pediatric Otolaryngology Department, Shenzhen Hospital, Southern Medical University, 518101 Shenzhen, Guangdong P. R. China

## Abstract

The vacuolar protein sorting 33B (VPS33B) was rarely reported in malignant tumors. In this research, we demonstrated that overexpression of VPS33B inhibited proliferation and chemoresistance to fluorouracil (5-FU) in nasopharyngeal carcinoma (NPC) in vivo and in vitro. Mechanistic analysis confirmed that overexpression of VPS33B modulated EGFR/PI3K/AKT/c-Myc/P53 signaling to arrest the cell cycle at G1/S phase. In addition, miR-133a-3p, a tumor-suppressive miRNA, was induced by P53 and directly targeted the EGFR/PI3K/AKT/c-Myc/P53 signaling and thus formed a negative feedback loop. Furthermore, another tumor suppressor, NESG1, interacted with VPS33B by colocalizing in the cytoplasm. The knockdown of NESG1 reversed the inhibitory effects of the overexpression of VPS33B in NPC cells by downregulating the PI3K/AKT/c-Jun-mediated transcription repression. Surprisingly, VPS33B was downregulated in the nicotine-treated and LMP-1-overexpressing NPC cells by targeting PI3K/AKT/c-Jun-mediated signaling. In addition, patients with higher VPS33B expression had a longer overall survival. Our study is the first to demonstrate that VPS33B is negatively regulated by LMP-1 and nicotine and thus suppresses the proliferation of NPC cells by interacting with NESG1 to regulate EGFR/PI3K/AKT/c-Myc/P53/miR-133a-3p signaling in NPC cells.

## Abstract (web summary)

VPS33B interacts with NESG1 to modulate EGFR/PI3K/AKT/c-Myc/P53/miR-133a-3p signaling and induce 5-Fluorouracil sensitivity in nasopharyngeal carcinoma, and VPS33B was inhibited by LMP-1 and nicotine.

## Graphical abstract

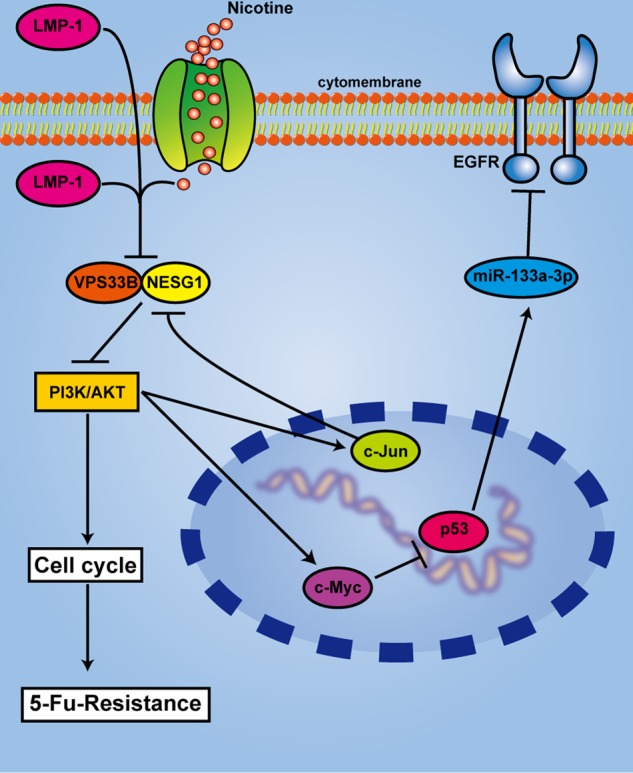

## Introduction

Nasopharyngeal carcinoma (NPC) is a malignant squamous cell carcinoma originating from epithelial cells in the nasopharynx, which is a leading cause of cancer death, especially in Southeast Asia/Middle East and North Africa^1^. In China, it is particularly endemic in the Guangdong and Guangxi provinces, with a characteristic of remarkable racial and geographic distribution and has threatened the health of numerous people in these areas^2–5^. Cigarette smoking causes direct damage to the nasal mucosa, which may promote the progression of rhinitis and even continuous nasopharyngeal (NP) damage and eventually stimulate the development of NPC^6,7^. Among the cigarette smoke components, nicotine is a pivotal factor in both the initiation of NPC carcinogenesis and progression^8,9^. Besides smoking and nicotine, Epstein–Barr virus (EBV) contributes to the carcinogenesis of NPC^10,11^. Latent membrane protein 1 (LMP-1) is an EBV oncogenic protein that disrupts NPC-related signaling^12,13^. However, the molecular mechanism of NPC pathogenesis is not yet clearly demonstrated. Despite the recent development in diagnostic technology and clinical strategies, advanced proliferation and distant metastasis remain major problems. Therefore, it is important to probe into the molecular basis of the development and progression of NPC, which contributes to identify better approaches to prevent and treat NPC.

The vacuolar protein sorting 33B (VPS33B) is a member of the Sec-1 domain family and encodes the human ortholog of rat Vps33b, which is homologous to the yeast class C Vps33 protein^14^. Mutations in this gene are associated with arthrogryposis–renal dysfunction–cholestasis syndrome^15^. Also, VPS33B plays a vital role in megakaryocyte biogenesis, platelet activation, and in vivo thrombosis and hemostasis^1,16^. In addition, VPS33B has been identified as a tumor suppressor in hepatocellular carcinoma (HCC)^17^.

NESG1 (also known as CFAP45 and CCDC19) encodes the cilia and flagella-associated protein 45, which is specially expressed in human nasopharynx and trachea^18,19^. We cloned and revised its coding sequence and preliminarily confirmed it as a potential tumor suppressor in NPC and non-small cell lung cancer (NSCLC) based on previous studies^20,21^.

Our previous researches have revealed that microRNAs (miRNAs) played vital roles in the carcinogenesis of several tumors. Our previous studies indicated that miR-3188 and miR-374a, respectively, target mammalian target of rapamycin and CCND1, which participate in the FOXO1- and PDCD4-stimulated inhibition of NPC growth, metastasis, and chemoresistance^22–24^. MiR-296-3p directly targets PRKCA, which participates in the HDGF/DDX5/CTNNB1/c-Myc-modulated network in lung adenocarcinoma^25^. In this research, we demonstrated that miR-133a-3p suppressed proliferation of NPC via modulation by the cooperation of VPS33B and NESG1.

In this study, we confirmed that VPS33B is downregulated in the nicotine-treated and LMP-1-overexpressing NPC cells through targeting phosphoinositide-3 kinase (PI3K)/AKT/c-Jun signaling. Moreover, the interaction of VPS33B with NESG1 suppresses the proliferation and the chemoresistance to fluorouracil (5-FU) in NPC by inactivating the epidermal growth factor receptor (EGFR)/PI3K/AKT/c-Myc/P53/miR-133a-3p feedback loop mechanism. Our findings provide, for the first time, a deeper understanding of the mechanism of the antiproliferative effect of VPS33B on NPC cells.

## Results

### VPS33B suppressed NPC cell growth and chemoresistance to 5-FU in vitro or in vivo and inactivated the EGFR/PI3K/AKT signaling

**Fig. 1:**
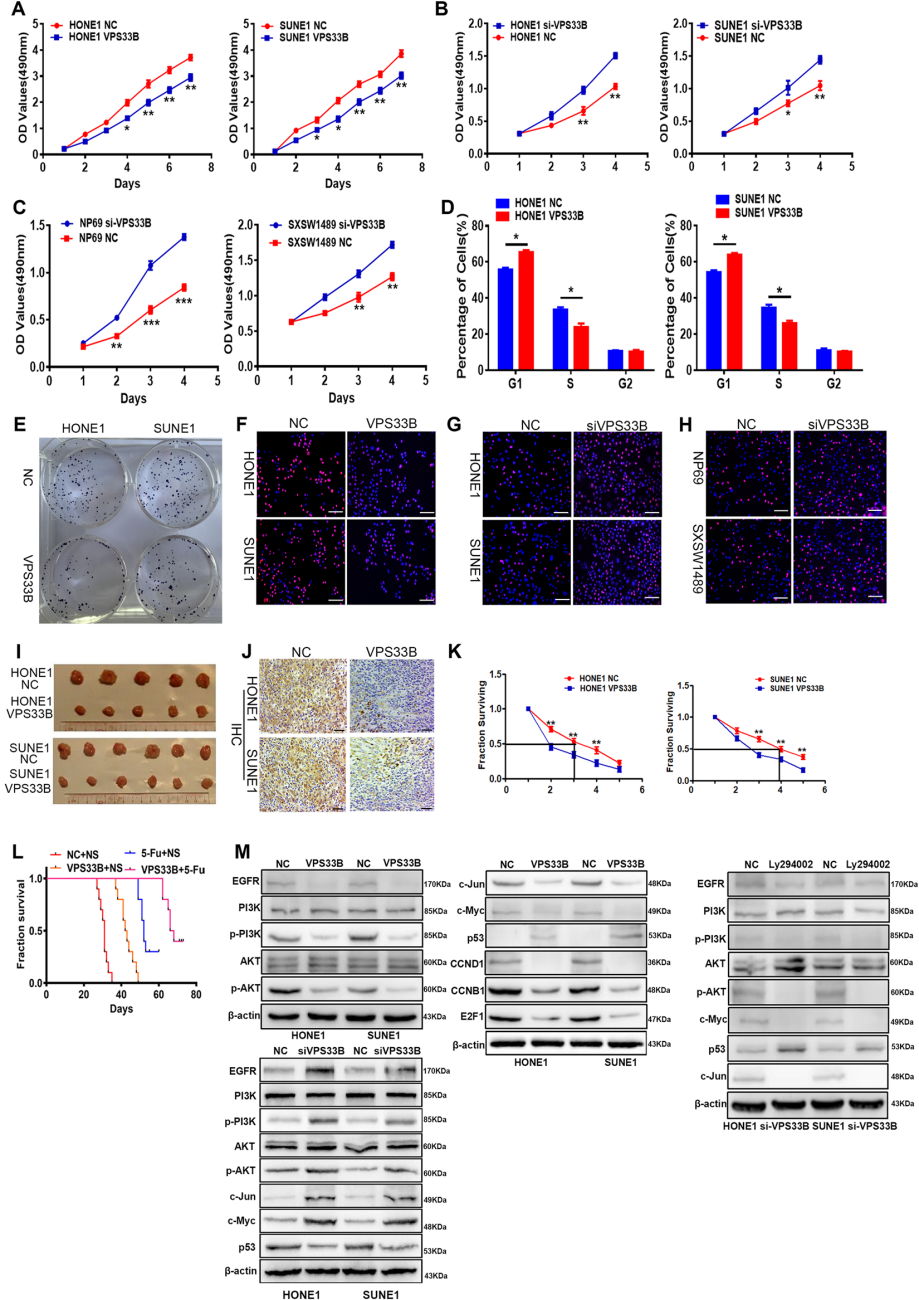
Vacuolar protein sorting 33B (VPS33B) suppressed nasopharyngeal carcinoma cell growth and chemoresistance to fluorouracil (5-FU) in vitro or in vivo and inactivated the epidermal growth factor receptor (EGFR)/phosphoinositide-3 kinase (PI3K)/AKT signaling. **a**–**h** 3-[4,5-Dimethylthiazol-2-yl]-2,5 diphenyl tetrazolium bromide assay (**a**–**c**), flow cytometry (**d**), colony-formation assay (**e**), and 5-ethynyl-2′-deoxyuridine assay (**f**–**h**) conducted to measure changes in HONE1 and SUNE or NP69 and SXSW1489 cell proliferation and cell cycle after transfection of Lv-GFP-VPS33B or small interfering RNA for VPS33B, respectively. Student’s *t* test, one-way analysis of varaince and Dunnett’s multiple comparison test. Mean ± SD, *P < 0.05; **P < 0.01. Scale bar: 100 μm. **i** Tumorigenicity of VPS33B-overexpressed HONE1 and SUNE1 cells was reduced in vivo, compared with negative control cells. **j** Representative proliferating cell nuclear antigen immunohistochemical staining of primary tumor tissues are shown. Scale bar: 50 μm. **k** Effects of VPS33B on 5-FU sensitivity to HONE1 and SUNE1 cells. **l** Survival analysis showed cumulative overall survival time ranked low to high, as follows: NC+NS<VPS33B+NS<NC+5-FU<VPS33B+5-FU, *n* = 10/group. Log-rank test, **P < 0.01. **m** Changes in EGFR, PI3K/AKT, p-PI3K/p-AKT, c-Jun, c-Myc, p53, CCNB1, CCND1, and E2F1 expression were detected by western blot analysis in HONE1 and SUNE1 or HONE1-siVPS33B and SUNE1-siVPS33B cells after transfection of Lv-GFP-VPS33B or PI3K inhibitor Ly294002, respectively. β-Actin was used as a loading control. All experiments were repeated three times

To study the biological functions of VPS33B, we ectopically expressed VPS33B in the NPC cell lines HONE1 and SUNE1 using a lentivirus. Quantitative polymerase chain reaction (qPCR) and western blot analysis showed that the mRNA and protein levels of the VPS33B in HONE1 and SUNE1 cells were increased compared with those in their respective negative control cells (Supplementary Figure 1A). We also studied the rate of cell proliferation in VPS33B-overexpressing HONE1 and SUNE1 cells. The growth curves determined by the 3-[4,5-dimethylthiazol-2-yl]-2,5 diphenyl tetrazolium bromide (<MTT) assay showed that overexpression of VPS33B suppressed cell proliferation in these two cell lines (Fig. 1a). The VPS33B-overexpressing HONE1 and SUNE1 cells also formed less colonies than their respective negative control cells in a colony-formation assay (Fig. 1e; Supplementary Figure 1F). In addition, we evaluated the cell cycle distribution of VPS33B-overexpressing HONE1 and SUNE1 cells and found that the G1-phase population was increased, while the S-phase population was decreased compared to their negative control cells using flow cytometry (Fig. 1d; Supplementary Figure 1G) and 5-ethynyl-2′-deoxyuridine (EdU) staining assay (Fig. 1f; Supplementary Figure 1H).

Additionally, NPC cell growth was evaluated in vivo by inoculating Lv-VPS33B-GFP HONE1 and SUNE1 cells into nude mice. The average xenograft tumor weight and volume of tumors derived from both cell lines overexpressing VPS33B were decreased compared with the respective negative control (Fig. 1i; Supplementary Figure 1K), while immunohistochemistry (IHC) of proliferating cell nuclear antigen and hematoxylin and eosin staining further confirmed that VPS33B exerted a suppressive effect on proliferation of NPC (Fig. 1j; Supplementary Figure 1L). Our results suggested a significant inhibitory effect of VPS33B on NPC proliferation both in vivo and in vitro.

To further confirm the biological function of VPS33B in nasopharynx, we used a small interfering RNA (siRNA) to specifically knock down VPS33B expression in NPC cells and normal epithelial cell lines NP69 and SXSW1489. Decreased expression of VPS33B was confirmed by qPCR and western blot (Supplementary Figure 1B-E). The results of the MTT and EdU staining assays confirmed that the downregulation of VPS33B increased the proliferation of NPC cells and NP cells (Fig. 1b, c, g, h; Supplementary Figure 1I-J).

Surprisingly, the NPC cell lines with stably overexpressing VPS33B exhibited significantly increased sensitivity to 5-FU. The inhibition rates after a 48-h treatment with different concentrations of 5-FU were determined. The half maximal inhibitory concentration (IC50) of 5-FU in HONE1 cells was reduced from 13.32 to 3.94 μM after transfection with VPS33B. A similar reduction in the IC50 from 14.45 to 5.21 μM occurred in VPS33B-overexpressing SUNE1 cells (Fig. 1k). This effect was further confirmed in vivo using VPS33B-overexpressing HONE1 and SUNE1 xenografts in nude mice. Survival times estimated by the Kaplan–Meier method confirmed that 5-FU treatment (NC+5-FU) or VPS33B overexpression (VPS33B+normal saline (NS)) alone extended survival compared to untreated normal controls (NC+NS). However, overexpression of VPS33B coupled with 5-FU treatment (VPS33B+5-FU) remarkably prolonged survival time beyond that of the other three groups (Fig. 1l). The median survival time for the NC+NS group was 31 days, while that of VPS33B+NS was 42.5 days and that of 5-FU+NS was 55 days, while that of VPS33B+5-FU was 67 days.

Western blot analysis showed that overexpression of VPS33B significantly reduced the expression of the EGFR and its downstream effectors phospho-PI3K and phospho-AKT. However, we did not observe a change in the total protein expression levels of PI3K/AKT (Fig. 1m). Furthermore, overexpression of VPS33B downregulated the expression of c-Myc, c-Jun, CCND1, CCNB1, and E2F1 were downregulated, while P53 was upregulated (Fig. 1m).

Moreover, we used siRNA to interfere the expression of VPS33B and introduced the PI3K/AKT signaling inhibitor Ly294002 to HONE1 and SUNE1 cells to further confirm whether overexpressed VPS33B could inactivate PI3K/AKT signaling pathway. We saw that Ly294002 reversed the activation of siRNA-VPS33B in related signaling in these cells (Fig. 1m).

### c-Myc/p53 inactivated the suppressive effect of miR-133a-3p in the proliferation of NPC cells via the modulation of the EGFR/PI3K/AKT signaling pathway

**Fig. 2:**
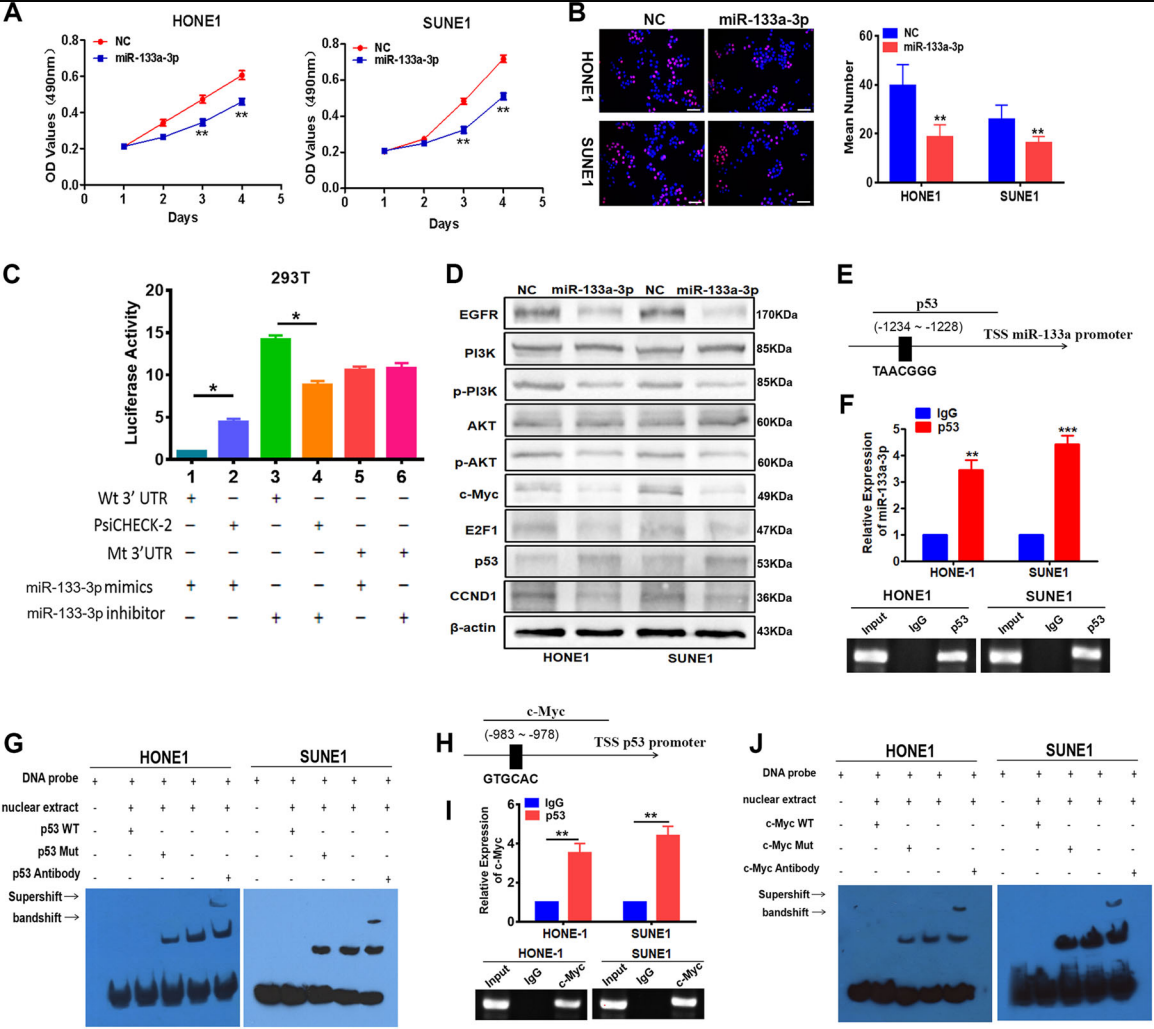
miR-133a-3p suppressed the proliferation of nasopharyngeal carcinoma cells via the modulation of the epidermal growth factor receptor (EGFR)/phosphoinositide-3 kinase (PI3K)/AKT signaling pathway. **a**, **b** 3-[4,5-Dimethylthiazol-2-yl]-2,5 diphenyl tetrazolium bromide (**a**) and 5-ethynyl-2′-deoxyuridine incorporation (**b**) assays were performed to demonstrate the impact of miR-133a-3p on the proliferation of A549 and H1975 cells. Student’s *t* test, one-way analysis of variance (ANOVA), mean ± SD, **P < 0.01. Scale bar: 100 μm. **c** That miR-133a-3p directly targeted EGFR was confirmed by luciferase reporter assay, one-way ANOVA, and Dunnett’s multiple comparison test. Mean ± SD, *P < 0.05. **d** Changes in EGFR, PI3K/AKT, p-PI3K/p-AKT, c-Jun, c-Myc, p53, CCND1, and E2F1 expression were detected by western blot analysis in HONE1 and SUNE1 cells after transfection of miR-133a-3p mimics. **e** Bioinformatics analysis revealed the promoter regions of miR-133a-3p with the putative p53 binding site. **f** Quantitative polymerase chain reaction (qPCR) and Gel electrophoresis confirmed the amplification of p53-binding sites after chromatin immunoprecipitation (ChIP) using antibody against p53. IgG antibody was used as the negative control. Student’s *t* test, mean ± SD, **P < 0.01, ***P < 0.001. **g** Electrophoretic mobility shift assay (EMSA) and supershift assay of p53 binding to miR-133a-3p promoter in HONE1 and SUNE1 cells. Labeled wild-type probe was incubated without (lane 1) or with (lane 4) cell nuclear proteins in the absence or presence of unlabeled probe (lanes 2–3). Unlabeled wild-type probe (lane 2) and mutant p53 probe (lane 3) were used to compete with p53 binding, each at 100-fold excess. Supershift assay (lane 5) was performed using an anti-p53 antibody. **h** Bioinformatics analysis revealed the promoter regions of p53 with the putative c-Myc binding site. **i** qPCR and Gel electrophoresis confirmed the amplification of c-Myc-binding sites after ChIP using antibody against c-Myc. IgG antibody was used as the negative control. Student’s *t* test, mean ± SD, **P < 0.01. **j** EMSA and supershift assay of c-Myc binding to p53 promoter in HONE1 and SUNE1 cells. Labeled wild-type probe was incubated without (lane 1) or with (lane 4) cell nuclear proteins in the absence or presence of unlabeled probe (lanes 2–3). Unlabeled wild-type probe (lane 2) and mutant c-Myc probe (lane 3) were used to compete with c-Myc binding, each at 100-fold excess. Supershift assay (lane 5) was performed using an anti-c-Myc antibody

We further investigated the function of miR-133a-3p in the proliferation of NPC. We overexpressed or silenced miR-133a-3p in HONE1 and SUNE1 cells with miR-133a-3p mimics or inhibitor, and the efficiency of transfection was examined by reverse transcriptase–qPCR (RT-qPCR; Supplementary Figure 2A, B). Analysis with the MTT and EdU assays demonstrated that overexpression of miR-133a-3p led to cell cycle arrest in NPC cells (Fig. 2a, b). MiR-133a-3p was reported to directly target EGFR in tumors^26–28^. In addition, we demonstrated that miR-133a-3p downregulated the expression of EGFR in NPC cells (Supplementary Figure 2C). The results of luciferase activity reporter assay confirmed that miR-133a-3p directly targeted EGFR (Fig. 2c).

Moreover, western blot analysis confirmed that miR-133a-3p could inactivate the EGFR/PI3K/AKT signaling pathway and suppress the expression of the downstream effectors c-Myc, CCND1, and E2F1, whereas the expression of P53 was upregulated (Fig. 2d).

To search for an upstream transcription factor of miR-133-3p, we used the online University of California, Santa Cruz (UCSC) genome browser (http://genome.ucsc.edu/) and the ALGGEN PROMO software (http://alggen.lsi.upc.es/cgi-bin/promo_v3/promo/promoinit.cgi?dirDB = TF_8.3) to find that P53 could bind to the miR-133a-3p promoter and precursor (Fig. 2e). RT-qPCR analysis revealed that the expression level of miR-133a-3p was decreased after silencing P53 (Supplementary Figure 2D). Also, chromatin immunoprecipitation (ChIP) and electrophoretic mobility shift assay (EMSA) confirmed that P53 directly bound to the miR-133a-3p promoter in NPC cells (Fig. 2f, g). Moreover, the results of the luciferase reporter activity assay validated that P53 activated miR-133a-3p transcription activity (Supplementary Figure 2E).

To further verify the role of the P53/miR-133a-3p axis in NPC, the miR-133a-3p inhibitor was transfected into P53-overexpressing HONE1 and SUNE1 cells and the expression of various proteins involved in the EGFR/PI3K/AKT/c-Myc signaling pathway was examined. The findings suggested the activation of EGFR/PI3K/AKT/c-Myc signaling and a decrease in P53 expression (Supplementary Figure 2F).

When c-Myc expression was disrupted (Supplementary Figure 2G), the expression of P53 and miR-133a-3p in NPC cells was upregulated (Supplementary Figure 2H, I). The results of the search for c-Myc-binding elements with the bioinformatic prediction software suggested that c-Myc could bind to the promoter of P53 (Fig. 2h). ChIP, EMSA analysis (Fig. 2i, j), and luciferase activity assays indicated that c-Myc directly bound to the P53 promoter and reduced its activity in NPC cells (Supplementary Figure 2J, K). In addition, the transduction of P53 in c-Myc-overexpressing NPC cells not only reduced cell growth and EdU staining (Supplementary Figure 2L, M) but also suppressed EGFR/PI3K/AKT/c-Myc signaling and upregulated miR-133a-3p expression (Supplementary Figure 2N, O).

Transfection of EGFR cDNA (pcDNA 3.1+EGFR cDNA) into HONE1 and SUNE1 cells (Supplementary Figure 3A) contributed to the activation of EGFR/PI3K/AKT/c-Myc signaling (Supplementary Figure 3B). In addition, the expression levels of miR-133a-3p were significantly downregulated (Supplementary Figure 3 C). The ChIP analysis results indicated that the binding of P53 to the miR-133a-3p promoter was reduced following EGFR overexpression (Supplementary Figure 3D).

### VPS33B modulated EGFR/PI3K/AKT/c-Myc/P53/miR-133a-3p signal

To determine whether miR-133a-3p participated in the VPS33B-modulated EGFR/PI3K/AKT/c-Myc/p53 signaling pathway and the formation of a loop mechanism in NPC cells, we examined the expression of miR-133a-3p in VPS33B-overexpressing cells by RT-qPCR analysis. The results indicated that VPS33B upregulated the expression of miR-133a-3p (Supplementary Figure 3E). Furthermore, transfection of EGFR cDNA not only increased cell growth and EdU staining (Supplementary Figure 3F-H) but also induced PI3K/AKT/c-Myc signaling and reduced the levels of P53 and miR-133a-3p in VPS33B-overexpressing cells (Supplementary Figure 3I, J). What is more, growth factors like EGF could stimulate the NPC cells to activate EGFR/PI3K/AKT/c-Jun signals (Supplementary Figure 3I). Meanwhile, with the transfection of the pcDNA3.1-EGFR plasmid, the binding of c-Myc with the P53 promoter was enhanced, while the binding of P53 with the miR-133a-3p promoter was reduced in VPS33B-overexpressing cells (Supplementary Figure 3K, L).

### VPS33B interacted with NESG1 through suppressing EGFR/PI3K/AKT/c-Jun pathway in NPC cells

**Fig. 3:**
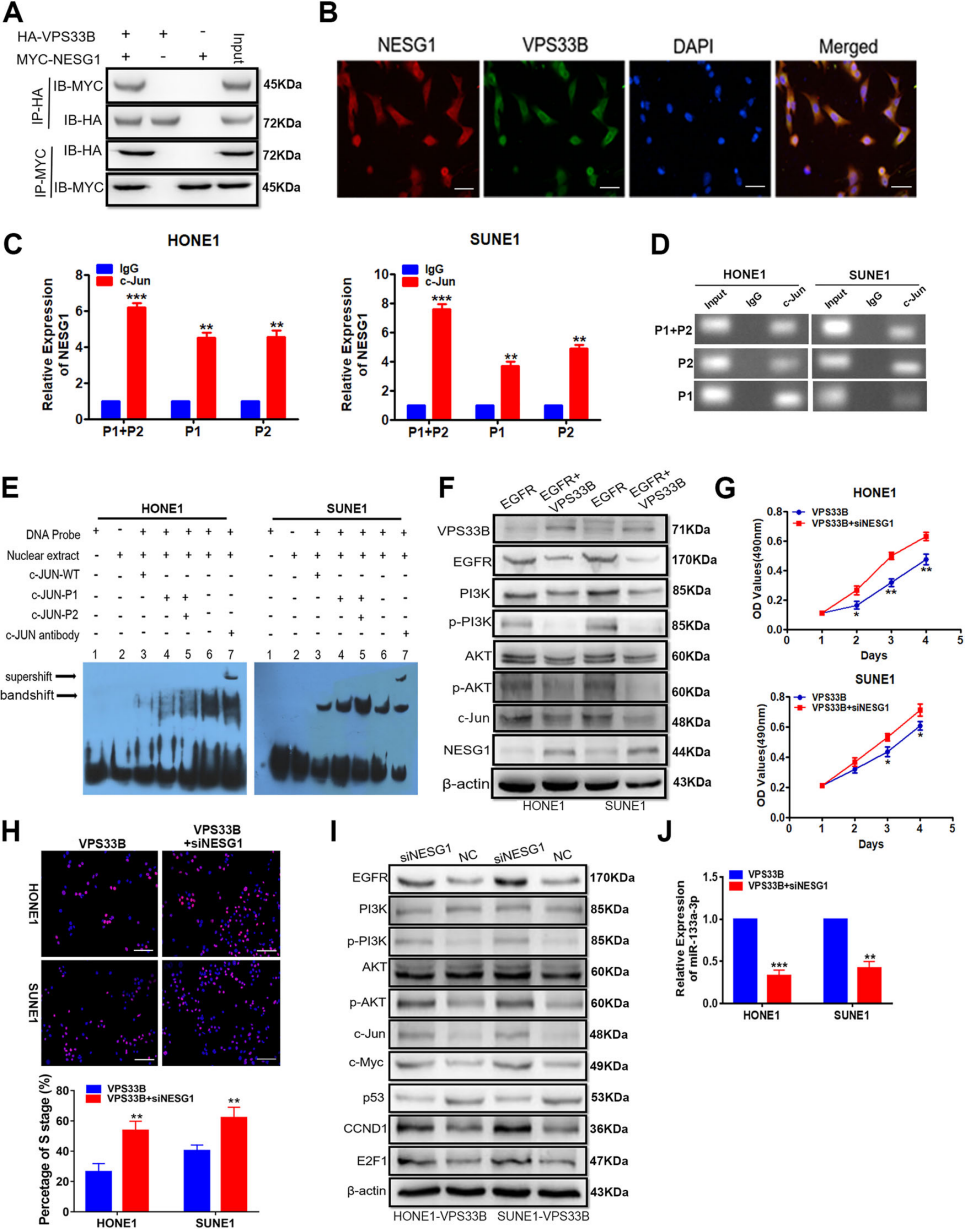
Vacuolar protein sorting 33B (VPS33B) interacted with NESG1 through suppressing epidermal growth factor receptor (EGFR)/phosphoinositide-3 kinase (PI3K)/AKT/c-Jun pathway in nasopharyngeal carcinoma cells. **a** Confirming the effectiveness of transfection of HA-VPS33B and MYC-NESG1 and the interaction of VPS33B and NESG1 after co-immunoprecipitation in 293T cells transfected with HA-VPS33B and MYC-NESG1 by western blot. **b** VPS33B co-located with NESG1 in cytoplasm by laser confocal assay. Scale bar: 50 μm. **c**, **d** Quantitative polymerase chain reaction (qPCR) (**c**) and Gel electrophoresis (**d**) confirmed the amplification of c-Jun-binding sites of NESG1 after chromatin immunoprecipitation using antibody against c-Jun. IgG antibody was used as the negative control. Student’s *t* test, mean ± SD, **P < 0.01, ***P < 0.001. **e** Electrophoretic mobility shift assay and supershift assay of c-Jun binding to NESG1 promoter in A549 and H1975 cells. Labeled wild-type probe was incubated without (lane 1) or with (lane 5) cell nuclear proteins in the absence or presence of unlabeled probe (lanes 2–4). Unlabeled wild-type probe (lane 2) and mutant c-Jun probe (lanes 3 and 4) were used to compete with c-Jun binding, each at 100-fold excess. Supershift assay (lane 6) was performed using an anti-c-Jun antibody. **f** Changes in EGFR, PI3K/AKT/p-PI3K/p-AKT, c-Jun, and NESG1 expression were detected by western blot analysis in VPS33B-overexpressed HONE1 and SUNE1 cells after transfection of EGFR plasmids. β-Actin was used as a loading control. **g**, **h** 3-[4,5-Dimethylthiazol-2-yl]-2,5 diphenyl tetrazolium bromide (**g**) and 5-ethynyl-2′-deoxyuridine incorporation (**h**) assays were performed to demonstrate the impact of silencing NESG1 on the proliferation of VPS33B-overexpressing HONE1 and SUNE1 cells. Student’s *t* test, one-way analysis of variance, mean ± SD, *P < 0.05, **P < 0.01. Scale bar: 100 μm. **i** Changes in EGFR, PI3K/AKT/p-PI3K/p-AKT, c-Jun, c-Myc, p53, CCND1, and E2F1 expression were detected by western blot analysis in VPS33B-overexpressed HONE1 and SUNE1 cells after silencing NESG1. β-Actin was used as a loading control. **j** miR-133a-3p was downregulated in the VPS33B-overexpressing HONE1 and SUNE1 cells after silencing NESG1 by qPCR assay. Student’s *t* test, mean ± SD, **P < 0.01, ***P < 0.001

In a previous study, we identified VPS33B as a potential interacting protein of NESG1 by using the yeast-two hybrid (Y2H) technique^29^. To verify this interaction, we transfected HONE1 cells with the VPS33B plasmid carrying the HA flag and the NESG1 plasmid carrying the MYC flag. The co-immunoprecipitation (Co-IP) assay results proved the interaction between VPS33B and NESG1 (Fig. 3a) in HONE1 cells. Confocal laser scanning microscopic analysis in HONE1 cells transfected with HA-VPS33B and MYC-NESG1 plasmids confirmed that both VPS33B and NESG1 interacted in the cytoplasm (Fig. 3b).

Overexpression of VPS33B resulted in the upregulation of NESG1 mRNA and protein expression in NPC cells (Supplementary Figure 4A, B). The UCSC genome browser and the ALGGEN PROMO software were used to screen for transcription factors that bound to the NESG1 promoter. c-Jun was revealed as a potential factor (Supplementary Figure 4C). When c-Jun was overexpressed, the expression of NESG1 was decreased relative to those of the control (Supplementary Figure 4D). ChIP and EMSA analysis verified the binding of c-Jun to the NESG1 promoter (Fig. 3c–e). Additionally, the luciferase report assay confirmed that c-Jun reduced NESG1 expression by reducing its transcription activity in NPC cells (Supplementary Figure 4E).

Transfection of the EGFR plasmid in VPS33B-overexpressed cells activated PI3K/AKT/c-Jun signaling (Fig. 3f), reduced the expression of NESG1 (Fig. 3f), increased the combination of c-Jun with NESG1 promoter (Supplementary Figure 4F), and reduced the expression of miR-133a-3p (Supplementary Figure 4G). These findings indicated that VPS33B stimulated NESG1 expression by attenuating the EGFR/PI3K/AKT/c-Jun pathway.

The knockdown of NESG1 restored cell growth in VPS33B-overexpressing NPC cells (Fig. 3g, h). Furthermore, we also found that VPS33B-regulated signaling were reversed and the expression of miR-133a-3p was downregulated after silencing NESG1 in VPS33B-overexpressing NPC cells (Fig. 3i, j).

**Fig. 4:**
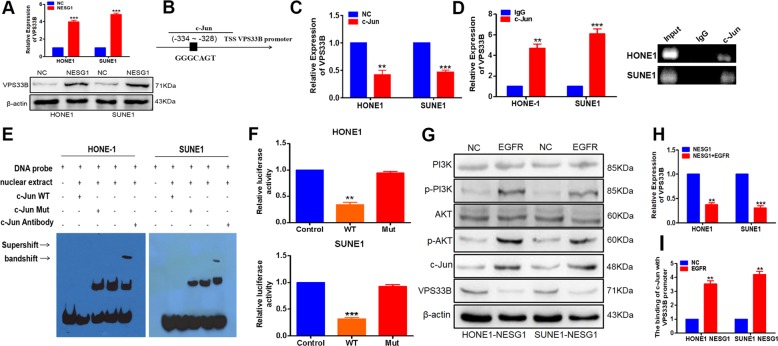
NESG1 stimulated the expression of vacuolar protein sorting 33B (VPS33B) via epidermal growth factor receptor (EGFR)/phosphoinositide-3 kinase (PI3K)/AKT/c-Jun signaling. **a** The mRNA and protein levels of VPS33B were upregulated in the NESG1-overexpressed HONE1 and SUNE1 cells by quantitative polymerase chain reaction (qPCR). Student’s *t* test, mean ± SD, *P < 0.05, **P < 0.01. **b** Bioinformatics analysis revealed the promoter regions of VPS33B with the putative c-Jun-binding site. **c** qPCR assay indicated that VPS33B was downregulated in the c-Jun-overexpressing HONE1 and SUNE1 cells. Student’s *t* test, mean ± SD, *P < 0.05, **P < 0.01. **d** qPCR and Gel electrophoresis confirmed the amplification of c-Jun-binding sites after chromatin immunoprecipitation using antibody against c-Jun. IgG antibody was used as the negative control. Student’s *t* test, mean ± SD, *P < 0.05, **P < 0.01. **e** Electrophoretic mobility shift assay and supershift assay of c-Jun binding to VPS33B promoter in HONE1 and SUNE1 cells. Labeled wild-type probe was incubated without (lane 1) or with (lane 4) cell nuclear proteins in the absence or presence of unlabeled probe (lanes 2–3). Unlabeled wild-type probe (lane 2) and mutant c-Jun probe (lane 3) were used to compete with c-Jun binding, each at 100-fold excess. Supershift assay (lane 5) was performed using an anti-c-Jun antibody. **f** Luciferase reporter assay demonstrated the luciferase activities of the wild type and Mut VPS33B promoter in HONE1 and SUNE1 cells transfected with c-Jun plasmid. Student’s *t* test, mean ± SD, *P < 0.05, **P < 0.01. **g** Changes in PI3K/AKT/p-PI3K/p-AKT, c-Jun, and VPS33B expression were detected by western blot analysis in NESG1-overexpressed HONE1 and SUNE1 cells after transduction of EGFR plasmids. β-Actin was used as a loading control. **h** qPCR assay indicated that VPS33B was downregulated in the NESG1-overexpressing HONE1 and SUNE1 cells after transduction of EGFR plasmids. Student’s *t* test, mean ± SD, *P < 0.05, **P < 0.01. **i** The binding of c-Jun with VPS33B promoter was enhanced in the NESG1-overexpressed HONE1 and SUNE1 cells after transfecting with EGFR plasmids. Student’s *t* test, mean ± SD, *P < 0.05, **P < 0.01, ***P < 0.001

Overexpression of NESG1 resulted in the increased expression of the VPS33B mRNA and protein levels in NPC cells (Fig. 4a). The online database were find that c-Jun was also considered a potential binding factor to VPS33B (Fig. 4b). When c-Jun was overexpressed in NPC cells, similar outcome like NESG1 was confirmed in NPC cells (Fig. 4c–f).

Transfection of the EGFR plasmid in NESG1-overexpressing cells led to increased PI3K/AKT/c-Jun expression, decreased VPS33B mRNA and protein expression levels (Fig. 4g, h), and increased the binding of c-Jun to the VPS33B promoter (Fig. 4i). These findings suggested that NESG1 induced VPS33B expression by inhibiting the EGFR/PI3K/AKT/c-Jun pathway.

### LMP-1 and nicotine suppressed VPS33B expression through PI3K/AKT/c-Jun signaling

**Fig. 5:**
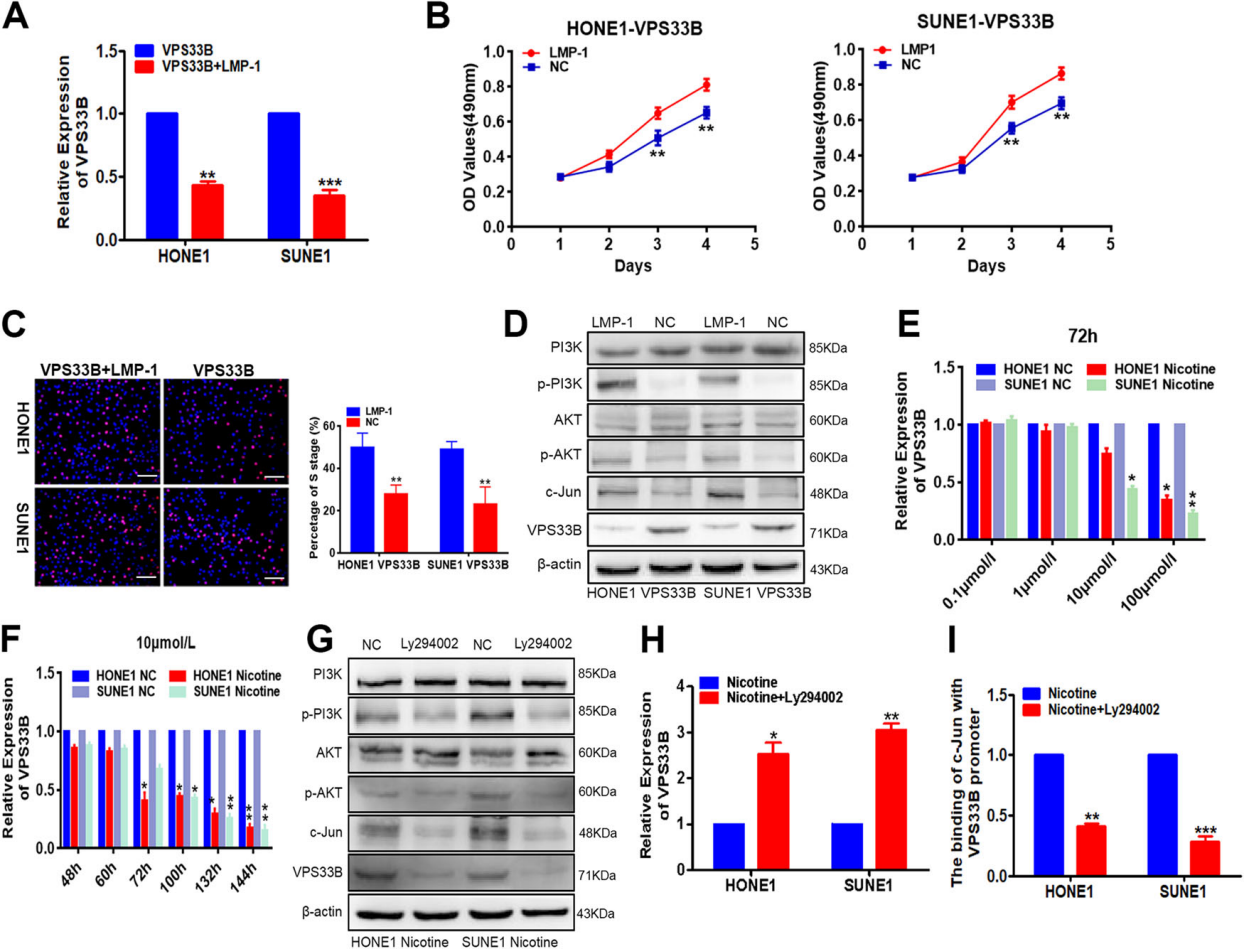
Latent membrane protein 1 (LMP-1) and nicotine suppressed vacuolar protein sorting 33B (VPS33B) expression through phosphoinositide-3 kinase (PI3K)/AKT/c-Jun signaling. **a** Quantitative polymerase chain reaction (qPCR) assay indicated that VPS33B was downregulated in the VPS33B-overexpressing HONE1 and SUNE1 cells after transduction of LMP-1 plasmids. Student’s *t* test, mean ± SD, *P < 0.05, **P < 0.01. **b**, **c** 3-[4,5-Dimethylthiazol-2-yl]-2,5 diphenyl tetrazolium bromide (**b**) and 5-ethynyl-2′-deoxyuridine incorporation (**c**) assays were performed to demonstrate the impact of miR-133a-3p on the proliferation of A549 and H1975 cells. Student’s *t* test, one-way analysis of variance, mean ± SD, *P < 0.05, **P < 0.01. Scale bar: 100 μm. **d** Changes in PI3K/AKT/p-PI3K/p-AKT and VPS33B expression were detected by western blot analysis in VPS33B-overexpressed HONE1 and SUNE1 cells after transduction of LMP-1 plasmids. β-Actin was used as a loading control. **e**, **f** The mRNA level of VPS33B were downregulated in the nicotine-treated HONE1 and SUNE1 cells in different concentrations (0.1, 1, 10, 100 μmol/L) at 72 h or at different times (24, 48, 72, 100, 132, and 132 h) in 10 μmol/L via qPCR assay. **g** Changes in PI3K/AKT/p-PI3K/p-AKT, c-Jun, and VPS33B expression were detected by western blot analysis in nicotine-treated A549 and H1975 cells after transfection of PI3K inhibitor Ly294002. β-Actin was used as a loading control. **h** VPS33B was indicated to be upregulated in nicotine-treated HONE1 and SUNE1 cells after transfection of PI3K inhibitor Ly294002 by qPCR assay. Student’s *t* test, mean ± SD, *P < 0.05, **P < 0.01. **i** The binding of c-Jun with VPS33B promoter was examined in nicotine-treated HONE1 and SUNE1 cells after transfecting Ly94002 by qPCR assay. Student’s *t* test, mean ± SD, *P < 0.05, **P < 0.01, ***P < 0.001

The mRNA and protein levels of VPS33B were downregulated in LMP-1-overexpressing HONE1 and SUNE1 cells (Fig. 5a, d). Furthermore, LMP-1 reversed the inhibitory effects of VPS33B on proliferation of NPC cells by MTT (Fig. 5b) and EdU incorporation assays (Fig. 5c). Additionally, LMP-1 positively modulated the PI3K/AKT/c-Jun signaling pathway in these cells (Fig. 5d). As mentioned above, c-Jun had a negative effect on the transcription of VPS33B by binding to its promoter. Here we reached the conclusion that LMP-1 negatively regulated VPS33B through the PI3K/AKT/c-Jun signaling pathway.

A further study of the effect in nicotine revealed that the mRNA levels of VPS33B was decreased in selected NPC cells treated with different concentrations of nicotine (0.1, 1, 10, and 100 μmol/L) for 72 h (Fig. 5e). Besides, we examined whether the mRNA levels of VPS33B were also downregulated in these NPC cells treated with 10 μmol/L nicotine for different times, including at 24, 48, 72, 100, 132, and 144 h (Fig. 5f).

Ultimately, the expression of VPS33B was found to be elevated in VPS33B-overexpressing NPC cells treated with nicotine after treatment with the PI3K specific inhibitor Ly294002 via PI3K/AKT/c-Jun signaling (Fig. 5g, h) and thus the binding of c-Jun with VPS33B promoter was facilitated (Fig. 5i).

### Clinicopathological characteristics of VPS33B expression in NPC

**Fig. 6:**
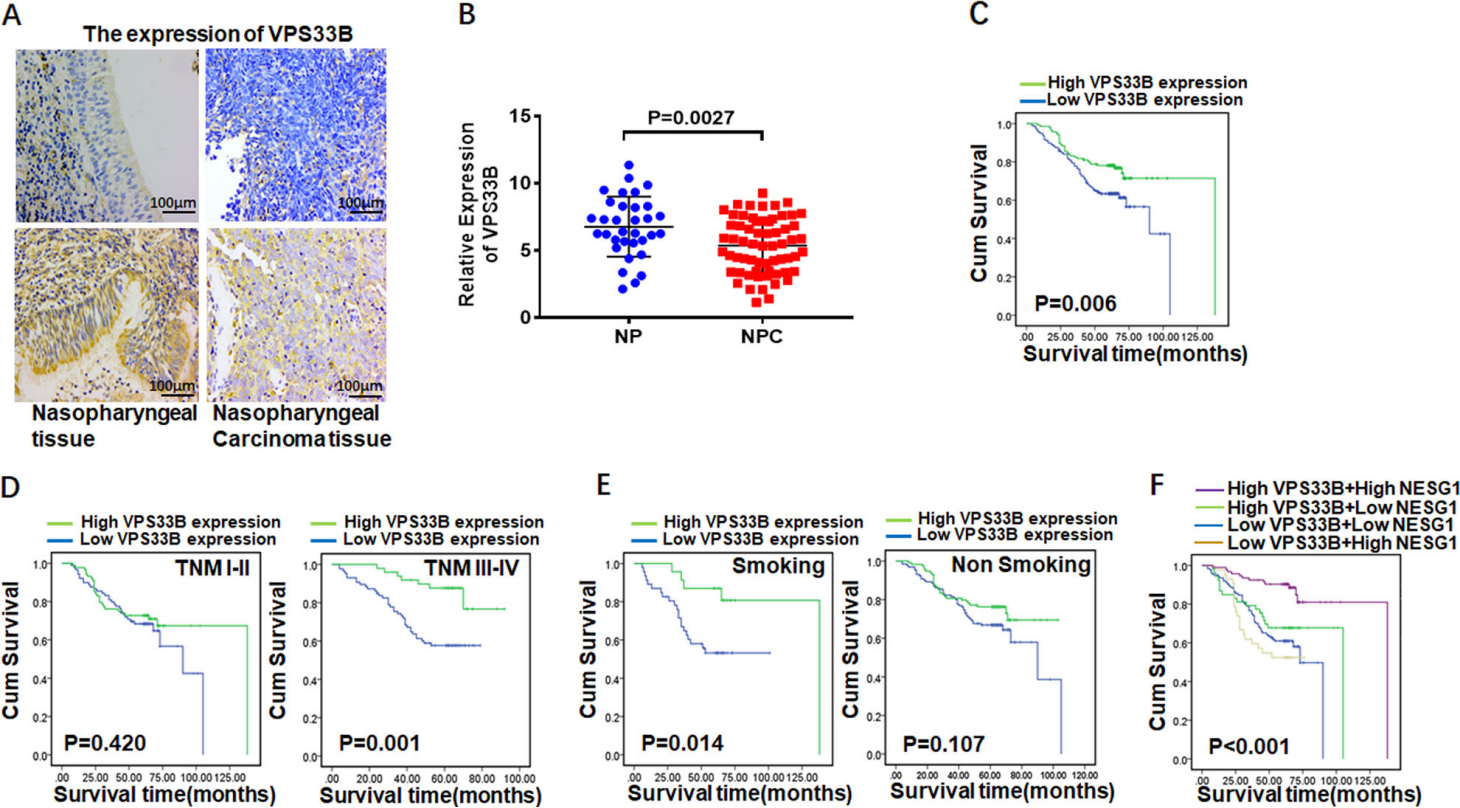
Vacuolar protein sorting 33B (VPS33B) contributed as a favorable prognosis factor in nasopharyngeal carcinoma (NPC). **a** Immunohistochemistry demonstrated that the protein expression of VPS33B was decreased in NPC tissues compared to their nasopharyngeal tissues. **b** VPS33B mRNA level was obviously increased in nasopharyngeal tissues compared to NPC tissues according to quantitative polymerase chain reaction analysis. **c** Kaplan–Meier survival analysis showed overall survival of NPC patients on the basis of VPS33B protein expression analysis. Log-rank test was used to calculate *P* values. **d** Stratified analysis, respectively, showed the survival prognosis of NPC patients in tumor node metastasis I–II or III–IV stage based on VPS33B protein expression analysis. **e** Stratified analysis, respectively, showed the survival prognosis of NPC patients in smoking or non-smoking status based on VPS33B protein expression analysis. **f** Kaplan–Meier survival analysis showed overall survival of NPC patients on the basis of NESG1 and VPS33B protein expression analysis. Log-rank test was used to calculate *P* values

We conducted IHC analysis in 313 NPC tissues and 134 NP tissues and RT-qPCR in 63 NPC tissues and 33 nasopharyngeal epithelial (NE) tissues to determine VPS33B protein and mRNA expression, respectively. The IHC staining results revealed the expression of cytoplasmic VPS33B in NPC and NP tissues (Table 1, Fig. 6a). We also found that the expression of VPS33B protein and mRNA in NPC tissues was significantly downregulated relative to that in NP tissues (*P* = 0.0026; Fig. 6b). The relationship between the clinicopathological characteristics and VPS33B expression in patients with NPC was summarized in Table 2 and Fig. 6c–f. VPS33B expression was negatively correlated with the TNM (tumor, node, metastasis) stage (*P* < 0.001; I–II vs. III–IV), M stage (*P* = 0.019; M0 vs. M1), and smoking (*P* = 0.048; Yes vs. No). Moreover, reduced VPS33B expression was significantly positively correlated with the overall survival time of the NPC patients (*P* = 0.006).

**Table 1:** The expression of VPS33B in NPC compared to non-cancerous nasopharyngeal tissues

Group	Cases (*n*)	VPS33B expression		*P* value
		High expression	Low expression	
NPC	313	137	176	**0.005**
Nasopharyngeal epithelium	134	78	56	

*NPC* nasopharyngeal carcinoma, *VPS33B* vacuolar protein sorting 33B

*P* values by χ^2^ test

Statistically significant values are in bold

**Table 2:** Correlations between VPS33B expression and the clinicopathological characteristics of NPC patients

Variables	VPS33B (%)			
	*N*	Low	High	*P* ^a^
Age, years				
<50	163	93 (57.1)	70 (42.9)	0.759
≥50	150	83 (55.3)	67 (44.7)	
Gender				
Male	98	53 (54.1)	44 (45.9)	0.704
Female	215	123 (57.2)	93 (42.8)	
TNM stage				
I+II	129	59 (50.3)	70 (49.7)	**<0.001**
III+IV	184	127 (63.9)	57 (36.1)	
T stage				
T1+T2	225	121 (53.8)	104 (46.2)	0.162
T3+T4	88	55 (62.5)	33 (37.5)	
N stage				
N0+N1	161	93 (57.8)	68 (42.2)	0.573
N2+N3	152	83 (54.6)	69 (45.4)	
M stage				
M0	289	157 (17.7)	132 (82.3)	**0.018**
M1	24	19 (79.2)	5 (20.8)	
Smoking				
Yes	69	46 (66.7)	23 (33.3)	**0.048**
No	244	130 (53.3)	114 (46.7)	
Family tumor history				
Yes	17	7 (41.2)	10 (58.8)	0.198
No	296	169 (57.1)	127 (42.9)	

*NPC* nasopharyngeal carcinoma, *TNM* tumor, node, metastasis, *VPS33B* vacuolar protein sorting 33B

^a^*P* values by χ^2^ test

Statistically significant values are in bold

In the subsequent analysis, VPS33B expression was positively correlated with NESG1 expression (Table 3). Survival analysis revealed cumulative overall survival time as follows: low VPS33B+high NESG1<low VPS33B+low NESG1<high VPS33B+low NESG1<high VPS33B+high NESG1 in NPC patients (Fig. 6g). Henceforth, high expression of VPS33B together with high NESG1 expression was associated with better prognosis for NPC patients. The correlation between the clinicopathological characteristics and VPS33B-NESG1 expression in NPC patients was summarized in Table 4. VPS33B-NESG1 expression was negatively correlated with the T stage (*P* = 0.036; T1+T2 vs. T3–T4) and M stage (*P* = 0.031; M0 vs. M1).

**Table 3:** Cross table about the VPS33B and NESG1 expression

Variables	NESG1		
	Low expression	High expression	*P* ^a^
VPS33B			
Low expression	124	42	**<0.001**
High expression	53	93	

*VPS33B* vacuolar protein sorting 33B

^a^*P* values by χ^2^ test

Statistically significant values are in bold

**Table 4:** Correlations between VPS33B and NESG1 expression and the clinicopathological characteristics of NPC patients

Factors	*N*	VPS33B-NESG1 expression (%)				*P* value
		LL	HL	LH	HH	
Gender						
Male	97	37	14	16	30	*P* = 0.695
Female	216	87	39	27	63	
Age (years)						
<50	163	62 (38)	29 (17.8)	20 (12.3)	52 (31.9)	*P* = 0.695
≥50	150	62 (41.3)	24 (16)	23 (15.3)	41 (27.3)	
TNM stage						
I–II	177	65 (36.7)	27 (15.3)	31 (17.5)	54 (30.5)	*P* = 0.119
III–IV	136	59 (43)	26 (19.1)	12 (8.8)	39 (28.7)	
T stage						
T_1_–T_2_	225	78 (34.7)	42 (18.7)	32 (14.2)	73 (32.4)	***P*** = **0.036**
T_3_–T_4_	88	46 (52.3)	11 (12.5)	11 (12.5)	20 (22.7)	
N stage						
N_0_–N_1_	161	68 (42.2)	26 (16.1)	21 (13)	46 (28.6)	*P* = 0.812
N_2_–N_3_	152	56 (36.8)	27 (17.8)	22 (14.5)	47 (30.9)	
M stage						
M_0_	289	112 (38.8)	46 (15.9)	38 (13.5)	92 (31.8)	***P*** = **0.031**
M_1_	24	12 (50)	7 (29.2)	4 (16.7)	1 (4.2)	
Smoking						
Yes	69	30 (43.5)	15 (21.7)	5 (7.2)	19 (27.5)	*P* = 0.222
No	244	94 (38.5)	38 (15.6)	38 (15.6)	74 (30.3)	
Family tumor history						
Yes	17	3 (17.6)	4 (23.5)	3 (17.6)	7 (41.2)	*P* = 0.302
No	296	121 (40.9)	49 (16.6)	40 (13.5)	86 (29.1)	

*P* values by χ^2^ test

*LL* low VPS33B and low NESG1 expression, *LH* low VPS33B and high NESG1 expression, *HL* high VPS33B and low NESG1 expression, *HH* high VPS33B and high NESG1 expression, *NPC* nasopharyngeal carcinoma, *TNM* tumor, node, metastasis, *VPS33B* vacuolar protein sorting 33B

Statistically significant values are in bold

## Discussion

VPS33B has been reported as a tumor suppressor only in HCC^17^, but the precise mechanism of its tumor-inhibitory effect remains unclear. In this study, we first determined that overexpression of VPS33B inhibited the proliferation of NPC cells both in vitro and in vivo. Additionally, we also found that VPS33B overexpression sensitized NPC cells to 5-FU both in vitro and in vivo. These data suggested a proliferation and chemoresistance inhibitory role for VPS33B in NPC.

It is well established that cell cycle progression is an important process in the promotion of tumor cell proliferation and the development of chemotherapeutic resistance to 5-FU^30,31^. The biological functions of VPS33B identified in this study shed new light into the mechanism of its role in NPC carcinogenesis. Our additional research confirmed that the VPS33B-induced cell growth suppression and cell cycle arrest were mainly due to the inhibition of the expression of positive cell cycle factors, such as c-Myc, CCND1, and E2F1, as well as the activation of the expression of the negative cell cycle regulator P53. Moreover, the downregulated expression of the oncogenic transcription factor c-Jun was also detected in VPS33B-overexpressing NPC cells.

The EGFR is a transmembrane protein that acts as a receptor for members of the epidermal growth factor family. The EGFR is a key factor that activates the PI3K signaling pathway and its continuous activation leads to upregulation of cell proliferation and thus promotes the pathogenesis of tumors^32–35^. In this study, we found that overexpression of VPS33B downregulated EGFR and inactivated its downstream PI3K/AKT pathway and therefore induced cell cycle transition in NPC. Taken together, these data suggested that VPS33B acted as a potential tumor suppressor that participated in the suppression of the EGFR/PI3K/AKT-induced cell cycle signaling cascade.

Recent studies underscored the role of microRNAs as pivotal mediators participating in the modulation network of various tumor-related genes in tumor pathogenesis^36–38^. As revealed by our previous studies, miRNAs made great contributions in the carcinogenesis of NPC and lung adenocarcinoma^9,22,23,25^. In previous studies, miR-133a has been reported to directly target EGFR and thus suppressed tumor cell growth and metastasis^26–28^. In this study, miR-133a-3p inhibited NPC cell growth and inactivated the EGFR/PI3K/AKT/c-Myc/p53 signals in NPC. Notably, we predicted the presence of P53-binding site in the promoter of miR-133-3p using the online PROMO database and UCSC genome browser. Subsequently, P53 was confirmed to directly induce miR-133a-3p expression by binding to its promoter based on the results from the ChIP analysis, EMSA, and luciferase activity report assay.

Furthermore, overexpression of P53 suppressed the EGFR/pPI3K/pAKT/c-Myc pathway through the induction of miR-133a-3p expression. In primary mouse embryo fibroblasts, c-Myc inhibits P53 degradation by inducing competitive binding with p14ARF^39^. This data suggested that the role of c-Myc was consistent with that of p53. Inversely, c-Myc acts as an oncogene that negatively regulates P53 expression in tumor^40^. Here we found that knocking down expression of c-Myc stimulated P53 expression, which suggested that c-Myc-suppressed P53 expression occurred at the transcriptional level. Noteworthy, c-Myc was predicted as a potential transcription factor that binds to the promoter of P53 via the above online database. We also confirmed that c-Myc inhibited the transcription of P53 by binding to its promoter and reducing the activity of the promoter. Furthermore, transduction of p53 in c-Myc-overexpressed NPC cells not only reduced cell growth and EdU staining but also suppressed EGFR/PI3K/AKT/c-Myc signaling and upregulated miR-133a-3p expression. We eventually observed that the suppression of EGFR reduced PI3K/AKT/c-Myc signaling and increased P53 and miR-133a-3p expression. Taken together, these data demonstrated a negative feedback loop mechanism among EGFR/PI3K/AKT/c-Myc/p53/miR-133a-3p in NPC.

We have also demonstrated that VPS33B regulated the EGFR/PI3K/AKT/c-Myc/p53 signaling in NPC. It was further observed that overexpression of VPS33B stimulated the expression of miR-133a-3p. Transfection of EGFR markedly activated the EGFR/PI3K/AKT/c-Myc signaling pathway and reduced the expression of the P53/miR-133a signaling in VPS33B-overexpressing NPC cells. In addition, the binding of c-Myc to the P53 promoter or the binding of P53 to the miR-133a-3p promoter was found to be, respectively, increased or decreased in VPS33B-overexpressing NPC cells after transduction with EGFR. These findings demonstrated that VPS33B regulated the EGFR/PI3K/AKT/c-Myc/P53/miR-133a-3p feedback loop mechanism in NPC.

In our previous reports, NESG1 was studied and identified as a tumor suppressor in NPC and NSCLC^20,21^. Importantly, we identified VPS33B as a potential interacting protein of NESG1 using the Y2H assay^29^. In the subsequent study, we confirmed that VPS33B interacted with NESG1 and colocalized in the cytoplasm of NPC cells. Furthermore, we observed VPS33B and NESG1 stimulated each other via PI3K/AKT/c-Jun signaling. Taken together, these results demonstrated that VPS33B interacted with NESG1 to modulate the EGFR/PI3K/AKT/c-Myc/P53/miR-133a-3p signaling pathway in suppressing NPC proliferation.

LMP-1, a key EBV-encoded oncogenic protein in NPC carcinogenesis^12,13^, was found to suppress VPS33B expression by inducing the EGFR/PI3K/AKT/c-Jun-mediated transcription suppression in NPC cells. Additionally, we also observed that LMP-1 reversed the inhibitory effects of VPS33B in NPC growth. These results demonstrated that VPS33B was disrupted by LMP-1 and participated in EBV-induced NPC.

Smoking is a major factor in the induction of the development of tumor pathogenesis, including NPC^6,7^, and nicotine is regarded as one of the key component of cigarettes^8,9^. In this study, we determined that the mRNA and protein levels of VPS33B were suppressed in nicotine-treated NPC cells. The evaluation of the mechanism indicated that nicotine stimulated the PI3K/AKT signaling pathway and upregulated the expression of the oncogenic transcription factor c-Jun. Notably, we also found that nicotine facilitated the binding of c-Jun with VPS33B promoter. The above-mentioned findings indicated that chemical carcinogens alone or combined with EBV disrupt the function of VPS33B in NPC pathogenesis.

In subsequent investigation, the clinical sample data were found to ultimately support the significance of VPS33B in NPC pathogenesis. RT-qPCR analysis of the expression of VPS33B mRNA levels revealed that it was markedly decreased in NPC tissues compared to the levels in NP tissues. IHC analysis indicated that VPS33B was expressed in the cytoplasm of NE and NPC cells. Consistent with mRNA expression data, VPS33B protein expression was clearly reduced in NPC compared to NE cells. In addition, reduced VPS33B expression was negatively correlated with the TNM stage, M stage, and smoking but positively associated with the overall survival time of the patients. Interestingly, VPS33B was also observed to be positively correlated with NESG1 expression. NPC patients with high VPS33B expression and high NESG1 expression obtained the longest overall survival time while those with low VPS33B expression and high NESG1 expression had the shortest overall survival time. Furthermore, VPS33B-NESG1 expression was negatively correlated with T stage or M stage. Our study demonstrated the significance of the VPS33B–NESG1 axis in modulating the pathogenesis of NPC.

Taken together, the results reveal that VPS33B acts as a tumor suppressor in NPC, which is negatively regulated by nicotine and LMP-1 and interacts with NESG1 to co-regulate the EGFR/PI3K/AKT/c-Myc/P53/miR-133a-3p feedback loop signaling and its downstream cell cycle regulatory factors. Accordingly, VPS33B may be a novel molecular therapeutic target for the development of a new treatment approach for NPC.

## Materials and methods

### Cell culture

Two NPC cell lines, namely, HONE1 and SUNE1, were obtained from the Cancer Research Institute of Southern Medical University, Guangzhou, China. Both cell lines were cultured in RPMI 1640 medium (HyClone, Logan, UT, USA) supplemented with 10% fetal bovine serum (FBS; ExCell, Shanghai, China). Both cell lines were grown in a humidified chamber with 5% CO_2_ at 37 °C.

### Collection of primary NPC and non-cancerous nasopharynx specimens and ethics statement

The 63 primary fresh NPC samples with TNM staging and 33 non-cancerous fresh NP samples used in the RT-qCPR assay were collected from the People’s Hospital of Zhongshan City, China at the time of diagnosis before any therapy. All these fresh samples were immediately preserved in liquid nitrogen. Three hundred and twenty-one paraffin-embedded NPC specimens and 134 paraffin-embedded nasopharynx tissues used in the IHC analysis were obtained from the People’s Hospital of Zhongshan City, Guangdong Province, China at the time of diagnosis before any therapy. The clinical processes were approved by the Ethics Committees of the People’s Hospital of Zhongshan City. Informed consent was obtained from all the patients. The pathological stage classification of all specimens was confirmed based on the Eighth edition of the American Joint Committee on Cancer staging system.

### Lentivirus production and infection

The HONE1 and SUNE1 cells were infected with a lentiviral expression vector carrying VPS33B and its flanking control sequence were constructed by GeneChem, Shanghai, China. Overexpressing cells with green fluorescent protein (GFP) signals were selected for further experiments by fluorescence-activated cell sorting flow cytometry. Protein and RNA from these cells were isolated, and the expression of VPS33B were measured by western blot analysis and RT-qPCR analysis.

### Transient transfection with siRNAs, plasmid, and miR-133a-3p mimics/inhibitor

The siRNAs for silencing VPS33B, NESG1, c-Myc, or hsa-miR-133a-3p mimics and the inhibitor (Supplementary Table) were designed and synthesized by Guangzhou RiboBio (RiboBio Co., Ltd., Guangzhou, China). Plasmids for P53, c-Jun, and LMP-1 were purchased from Vigene Biosciences Inc. (Jinan City, China). Ly294002 was purchased from Sigma-Aldrich Inc. (Saint Louis, MO, USA). Cells were plated onto a 6-well plate or 96-well plate (Nest Biotech, China) at 30–50% confluence, 24 h before transfection. Plasmid constructs were then transfected into cells using Lipofectamine TM 2000 (Invitrogen Biotechnology, Guangzhou, China) according to the manufacturer’s protocol. Cells were collected after 24–72 h for further experiments. The sequences of each siRNA, mimics or inhibitor are listed in Supplementary Table 1

### RNA isolation, reverse transcription, and qRT-PCR

RNA was isolated from NPC cell lines, NPC tissues, and normal nasopharynx tissues using the Trizol reagent (TAKARA, Co., Ltd., Dalian, China) according to the instructions of the manufacturer and used for reverse transcription and qRT-PCR. Specific primers for VPS33B, miR-133a-3p, c-Myc, P53, EGFR, and NESG1 are shown in Supplementary Tables. The sequences of each primer used in the qPCR assay are listed in Supplementary Table 2.

### Cell proliferation analysis

The cell proliferation rate was determined using the MTT assay. NPC cells (1000/well) were seeded in 96-well plates. Afterwards, 20 µL of MTT (5 mg/ml; Sigma-Aldrich) was added to each well and cultured at 37 °C for 4 h. Subsequently, supernatants were removed and 150 μL of dimethyl sulfoxide (DMSO; Sigma-Aldrich) was added to measure the OD value of each well at 490 nm. For lentivirus-mediated VPS33B overexpression, the cells were incubated for 7 days. For transient transfection with si-NESG1, siVPS33B, miR-133a-3p mimics, miR-133a-3p inhibitor, etc., the cells were cultured for 4 days. MTT was used to analyze cell proliferation. Experiments were performed three times.

### Colony formation

Cells were seeded in 6-well culture plates at 200 cells/well. After incubation for 14 days, cells were washed twice with D-Hank’s solution and stained with a hematoxylin solution. The number of colonies containing ≥50 cells was counted under a microscope. All experiments were repeated at least three times.

### EdU incorporation

EdU incorporation analysis was performed using the Apollo567 In Vitro Imaging Kit (RiboBio Inc.) according to the manufacturer’s protocol. Cells were incubated with 10 μM EdU for 2 h before fixation with 4% paraformaldehyde, permeabilization with 0.3% Triton X-100, and staining with the Apollo fluorescent dyes. Cell nuclei were stained with 5 μg/ml DAPI (4’,6-diamidino-2-phenylindole) for 10 min. The number of EdU-positive cells were counted under a microscope in five random fields. All assays were independently performed at least three times.

### In vivo tumorigenesis assay

A total of 5 × 10^6^ logarithmically growing cells in 0.1 ml Hank’s solution were subcutaneously inoculated into the left–right symmetric flank of 4–6-week-old female BALB/c-nu/nu mice (*N* = 6 per group). Mice were maintained in a barrier facility on HEPA-filtered racks and fed an autoclaved laboratory rodent diet. All animal studies were conducted in accordance with the principles and procedures outlined in the Southern Medical University Guide for the Care and Use of Animals under assurance number SCXK (Guangdong) 2008–0002. Mice were sacrificed after 15 days and tumors were examined, weighed, and processed for histology.

### In vitro and in vivo 5-FU treatment experiment on nude mice

Drug sensitivity test was determined by the MTT assay. Cells were seeded in 96-well plates at a density of 2 × 10^3^ cells/well and treated with 0, 1, 2, 4, 8, or 16 µM 5-FU (Qilu Pharmaceutical Co., Ltd., Jinan City, China) for 48 h. Subsequently, 20 µL of MTT (5 mg/ml; Sigma-Aldrich) was added to each well and incubated at 37 °C for 4 h. Then supernatants were removed and 150 μL of DMSO (Sigma-Aldrich) was added to measure the absorbance value (OD) of each well at 490 nm. The calculated rates were used for curve fitting and calculation of IC50. Experiments were performed three times.

In all, 6 × 10^5^ VPS33B overexpressing HONE1 cells or their controls were intraperitoneally injected into 14 g female nu/nu mice (*N* = 24 each). Tumors were allowed to grow for 3 days and then animals were randomized into NC+NS (normal saline), NC+5-FU, VPS33B+NS, and VPS33B+5-FU for therapy testing. Survival curves were analyzed using Kaplan–Meier analysis.

### Western blot analysis

Western blot analysis was performed as described in a previous study. Antibodies included anti-VPS33B, EGFR, p-PI3K, PI3K, p-AKT, AKT, c-Jun, c-Myc, P53, p-Rb, E2F1, CCND1, CDK4, and β-actin antibody (1:1000; CW Biotechnology). Images were captured with a Chem iDoc^TM^CRS+ Molecular Imager (Bio-Rad Laboratories, Hercules, CA, USA). The details of each antibody used in the western blot are listed in Supplementary Table 3.

### IHC analysis

Paraffin sections (4 μm) from samples were deparaffinized and antigen retrieval was performed in citrate buffer for 3 min at 100 °C. Endogenous peroxidase activity and non-specific antigens were blocked with peroxidase blocking reagent followed by incubation with the appropriate antibody overnight at 4 °C. Antibody dilutions and sources are shown in Supplementary Table. After washing, sections were incubated with a biotin-labeled secondary antibody and subsequently incubated with streptavidin-conjugated horseradish peroxidase. The peroxidase reaction was developed using the 3,3-diaminobenzidine (DAB) chromogen solution in DAB buffer substrate (Maixin, Fuzhou, China). Sections were visualized with DAB and counterstained with hematoxylin, mounted in neutral gum, and analyzed using a brightfield microscope. The details of each antibody used in the IHC are listed in Supplementary Table 3.

### Luciferase activity report assays

EGFR was predicted to be a direct target of miR-133a-3p by the RNAhybrid and TargetScan software. A fragment of the EGFR 3’-untranslated region (3’-UTR) (wild-type 3’-UTR) was amplified. Site-directed mutagenesis (mut) of the miR-133a-3p-binding site was performed using the GeneTailor Site-Directed Mutagenesis System (Invitrogen Biotechnology). The wt 3’-UTR or mut 3’-UTR were cloned into psiCHECK-2 vectors. For luciferase reporter assays, the wt or mut 3’-UTR vector was co-transfected with the miR-133a-3p mimics/inhibitor or non-specific control into HONE1 cells. Luciferase activity was measured 48 h after transfection using the Dual-Luciferase Reporter Assay System (Promega Corporation, Madison, WI, USA). To examine the effect of P53 on the miR-133a-3p promoter activity, a fragment containing the two p53-binding sites was cloned into the pGL3-Basic luciferase reporter vector, and the p53-binding site mutation vectors were constructed. These vectors and P53 plasmid were co-transfected into HONE1 and SUNE1 cells. Luciferase activity of the miR-133a-3p promoter was measured 48 h after transfection. The sequences of the primers used in the luciferase activity report assay are listed in Supplementary Table 4.

### ChIP analysis

The UCSC and PROMO database analysis predicted the putative P53-binding sites on the miR-133a-3p promoter region. DNA–protein complexes were immunoprecipitated from HONE1 cells using the Chromatin Immunoprecipitation Kit (Thermo Fisher Scientific, Waltham, MA, USA) according to the manufacturer’s protocol using anti-P53 or IgG (Cell Signaling Technology, Danvers, MA, USA) antibodies. IgG served as a control for non-specific DNA binding. RT-qPCR analysis and PCR analysis were used to measure the enrichment of the miR-133a-3p promoter region. ChIP of c-Myc with P53, c-Jun with NESG1, and c-Jun with VPS33B were carried out in the similar procedures. The primers used in the ChIP assay are listed in Supplementary Table 2.

### Electrophoretic mobility shift assay

The binding of P53 to the miR-133a-3p promoter was detected using an EMSA Kit (Roche Diagnostics, Basel, Switzerland) according to the manufacturer’s instructions. The probe sequences are shown in Supplementary Table. The 3’-end of the wild-type probe was labeled with biotin. Samples without nucleoprotein were used as negative controls. The binding mixture included 5 μg of nuclear extracts and 1 μg poly (dI:dC) incubated in the presence or absence of 100-fold specific oligonucleotide competitor (unlabeled wild-type or mutant P53 probe) for 15 min at room temperature before the addition of the biotin-labeled wild-type probe. Signals were recorded using a BioSens Gel Imaging System (BIOTOP Co., Ltd., Shanghai, China). EMSA analysis was performed at Biosense Bioscience Co. Ltd., (Guangzhou, China). EMSA of c-Myc with P53, c-Jun with NESG1, and c-Jun with VPS33B were carried out in the similar procedures. The sequences of the probes used in the EMSA assay are listed in Supplementary Table 5.

### Co-immunoprecipitation

Co-IP was performed using a Pierce Co-Immunoprecipitation Kit (Thermo Fisher scientific) according to the manufacturer’s instructions. Briefly, total protein was extracted and quantified. A total of 3000 μg of protein in 500 μL supernatant was incubated with 10 μg anti-HA, anti-MYC, or anti-IgG antibodies for 12 h at 4 °C. Immune complexes were stained with Coomassie brilliant blue staining and analyzed by western blot analysis. Anti-IgG was used as a negative control.

### Nicotine-treated related assays

Nicotine was dissolved in DMSO and subsequently added to selected NPC cells at concentrations of 0.1, 1, 10, or 100 μmol/L, and these cells were cultured in RPMI 1640 medium (HyClone) supplemented with 10% FBS (ExCell, Shanghai, China) for different periods of time, as described above. The nicotine-treated cells were collected to perform the related in vitro assays by the above described methods.

### Statistical analysis

Statistical analyses were performed with the SPSS 16.0 statistical software package (SPSS Inc., Chicago, IL, USA). Data are expressed as mean ± SD from at least three independent experiments. Differences were evaluated by Student’s *t* test for two groups, one-way analysis of variance for multiple groups, and parametric generalized linear model with random effects for tumor growth and MTT assay and were considered to be statistically significant at values of *P* < 0.05. Analysis of the data of VPS33B expression in primary NPC tissues and normal NP tissues was performed using paired-samples *T* test. Relationships were analyzed by Spearman’s correlation analysis. Survival analysis was performed using the Kaplan–Meier method. All statistical tests were two-sided, and asterisks indicate statistical significance.

## Supplementary information

Supplementary Figure 1

Supplementary Figure 2

Supplementary Figure 3

Supplementary Figure 4

Supplementary Table 1

Supplementary Table 2

Supplementary Table 3

Supplementary Table 4

Supplementary Table 5

supplemental figure legends

## References

[1] Zhou Y, Zhang J (2014). Arthrogryposis-renal dysfunction-cholestasis (ARC) syndrome: from molecular genetics to clinical features. Ital. J. Pediatr..

[2] Petersson F (2015). Nasopharyngeal carcinoma: a review. Semin. Diagn. Pathol..

[3] Lin K (2018). Lack of association between the distribution of ABO blood groups and nasopharyngeal carcinoma in a population of Southern China. J. Cancer Res. Ther..

[4] Tang LQ (2018). Concurrent chemoradiotherapy with nedaplatin versus cisplatin in stage II-IVB nasopharyngeal carcinoma: an open-label, non-inferiority, randomised phase 3 trial. Lancet Oncol..

[5] Coghill AE (2018). Identification of a novel, EBV-based antibody risk stratification signature for early detection of nasopharyngeal carcinoma in Taiwan. Clin. Cancer Res..

[6] Chen C, Shen LJ, Li BF, Gao J, Xia YF (2014). Smoking is a poor prognostic factor for male nasopharyngeal carcinoma treated with radiotherapy. Radiother. Oncol..

[7] Guo SS (2014). The impact of smoking on the clinical outcome of locoregionally advanced nasopharyngeal carcinoma after chemoradiotherapy. Radiat. Oncol..

[8] Shi D (2012). Nicotine promotes proliferation of human nasopharyngeal carcinoma cells by regulating alpha7AChR, ERK, HIF-1alpha and VEGF/PEDF signaling. PLoS ONE.

[9] Deng X (2018). miR-296-3p negatively regulated by nicotine stimulates cytoplasmic translocation of c-Myc via MK2 to suppress chemotherapy resistance. Mol. Ther..

[10] Huang D (2017). Epstein-Barr virus-induced VEGF and GM-CSF drive nasopharyngeal carcinoma metastasis via recruitment and activation of macrophages. Cancer Res..

[11] Cai LM (2015). EBV-miR-BART7-3p promotes the EMT and metastasis of nasopharyngeal carcinoma cells by suppressing the tumor suppressor PTEN. Oncogene.

[12] Lu J (2016). EBV-LMP1 suppresses the DNA damage response through DNA-PK/AMPK signaling to promote radioresistance in nasopharyngeal carcinoma. Cancer Lett..

[13] He X (2016). Chromatin remodeling factor LSH drives cancer progression by suppressing the activity of fumarate hydratase. Cancer Res..

[14] Carim L, Sumoy L, Andreu N, Estivill X, Escarceller M (2000). Cloning, mapping and expression analysis of VPS33B, the human orthologue of rat Vps33b. Cytogenet. Cell Genet..

[15] Rosales A (2018). Severe renal Fanconi and management strategies in arthrogryposis-renal dysfunction-cholestasis syndrome: a case report. BMC Nephrol..

[16] Xiang B (2015). Characterization of a novel integrin binding protein, VPS33B, which is important for platelet activation and in vivo thrombosis and hemostasis. Circulation.

[17] Wang C (2018). Vacuolar protein sorting 33B is a tumor suppressor in hepatocarcinogenesis. Hepatology.

[18] Li Z, Yao K, Cao Y (1999). Molecular cloning of a novel tissue-specific gene from human nasopharyngeal epithelium. Gene.

[19] Liu Z (2011). Decreased expression of updated NESG1 in nasopharyngeal carcinoma: its potential role and preliminarily functional mechanism. Int. J. Cancer.

[20] Liu Z (2011). Potential tumor suppressor NESG1 as an unfavorable prognosis factor in nasopharyngeal carcinoma. PLoS ONE.

[21] Liu Z (2014). Candidate tumour suppressor CCDC19 regulates miR-184 direct targeting of C-Myc thereby suppressing cell growth in non-small cell lung cancers. J. Cell. Mol. Med..

[22] Zhao M (2016). miR-3188 regulates nasopharyngeal carcinoma proliferation and chemosensitivity through a FOXO1-modulated positive feedback loop with mTOR-p-PI3K/AKT-c-JUN. Nat. Commun..

[23] Zhen Y (2017). miR-374a-CCND1-pPI3K/AKT-c-JUN feedback loop modulated by PDCD4 suppresses cell growth, metastasis, and sensitizes nasopharyngeal carcinoma to cisplatin. Oncogene.

[24] Zhao M (2018). Dual roles of miR-374a by modulated c-Jun respectively targets CCND1-inducing PI3K/AKT signal and PTEN-suppressing Wnt/beta-catenin signaling in non-small-cell lung cancer. Cell Death Dis..

[25] Fu Q (2017). miRomics and proteomics reveal a miR-296-3p/PRKCA/FAK/Ras/c-Myc feedback loop modulated by HDGF/DDX5/beta-catenin complex in lung adenocarcinoma. Clin. Cancer Res..

[26] Yang QS, Jiang LP, He CY, Tong YN, Liu YY (2017). Up-regulation of microRNA-133a inhibits the MEK/ERK signaling pathway to promote cell apoptosis and enhance radio-sensitivity by targeting EGFR in esophageal cancer in vivo and in vitro. J. Cell. Biochem..

[27] Wei W, Liu Y, Lu Y, Yang B, Tang L (2017). LncRNA XIST promotes pancreatic cancer proliferation through miR-133a/EGFR. J. Cell. Biochem..

[28] Song X, Shi B, Huang K, Zhang W (2015). miR-133a inhibits cervical cancer growth by targeting EGFR. Oncol. Rep..

[29] Wang, H. *Screening, Identification and Functional Mechanisms of NESG1 Interacting Proteins*. Dissertation, Southern Medical Univ. (2014).

[30] Bendris N, Lemmers B, Blanchard JM (2015). Cell cycle, cytoskeleton dynamics and beyond: the many functions of cyclins and CDK inhibitors. Cell Cycle.

[31] Zhou R (2018). Histone deacetylase inhibitor AR-42 inhibits breast cancer cell growth and demonstrates a synergistic effect in combination with 5-FU. Oncol. Lett..

[32] Sun S (2018). STAT3/HOTAIR signaling axis regulates HNSCC growth in an EZH2-dependent manner. Clin. Cancer Res..

[33] Kamekura R (2014). Loss of the desmosomal cadherin desmoglein-2 suppresses colon cancer cell proliferation through EGFR signaling. Oncogene.

[34] Xia H (2018). EGFR-PI3K-PDK1 pathway regulates YAP signaling in hepatocellular carcinoma: the mechanism and its implications in targeted therapy. Cell Death Dis..

[35] Xu S (2018). LZTS2 inhibits PI3K/AKT activation and radioresistance in nasopharyngeal carcinoma by interacting with p85. Cancer Lett..

[36] Rui X (2018). Long non-coding RNA C5orf66-AS1 promotes cell proliferation in cervical cancer by targeting miR-637/RING1 axis. Cell Death Dis..

[37] Liu H (2019). GATA6 suppresses migration and metastasis by regulating the miR-520b/CREB1 axis in gastric cancer. Cell Death Dis..

[38] Pu W (2018). Targeting Pin1 by inhibitor API-1 regulates microRNA biogenesis and suppresses hepatocellular carcinoma development. Hepatology.

[39] Madapura HS (2016). cMyc-p53 feedback mechanism regulates the dynamics of T lymphocytes in the immune response. Cell Cycle.

[40] Eischen CM, Weber JD, Roussel MF, Sherr CJ, Cleveland JL (1999). Disruption of the ARF-Mdm2-p53 tumor suppressor pathway in Myc-induced lymphomagenesis. Genes Dev..

