# Supplementary figures and images for: VPS33B interacts with NESG1 to modulate EGFR/PI3K/AKT/c-Myc/P53/miR-133a-3p signaling and induce 5-fluorouracil sensitivity in nasopharyngeal carcinoma
Source: Cell Death Dis. 2019 Apr 3;10(4):305. doi: 10.1038/s41419-019-1457-9 (PMC6447525; doi:10.1038/s41419-019-1457-9)

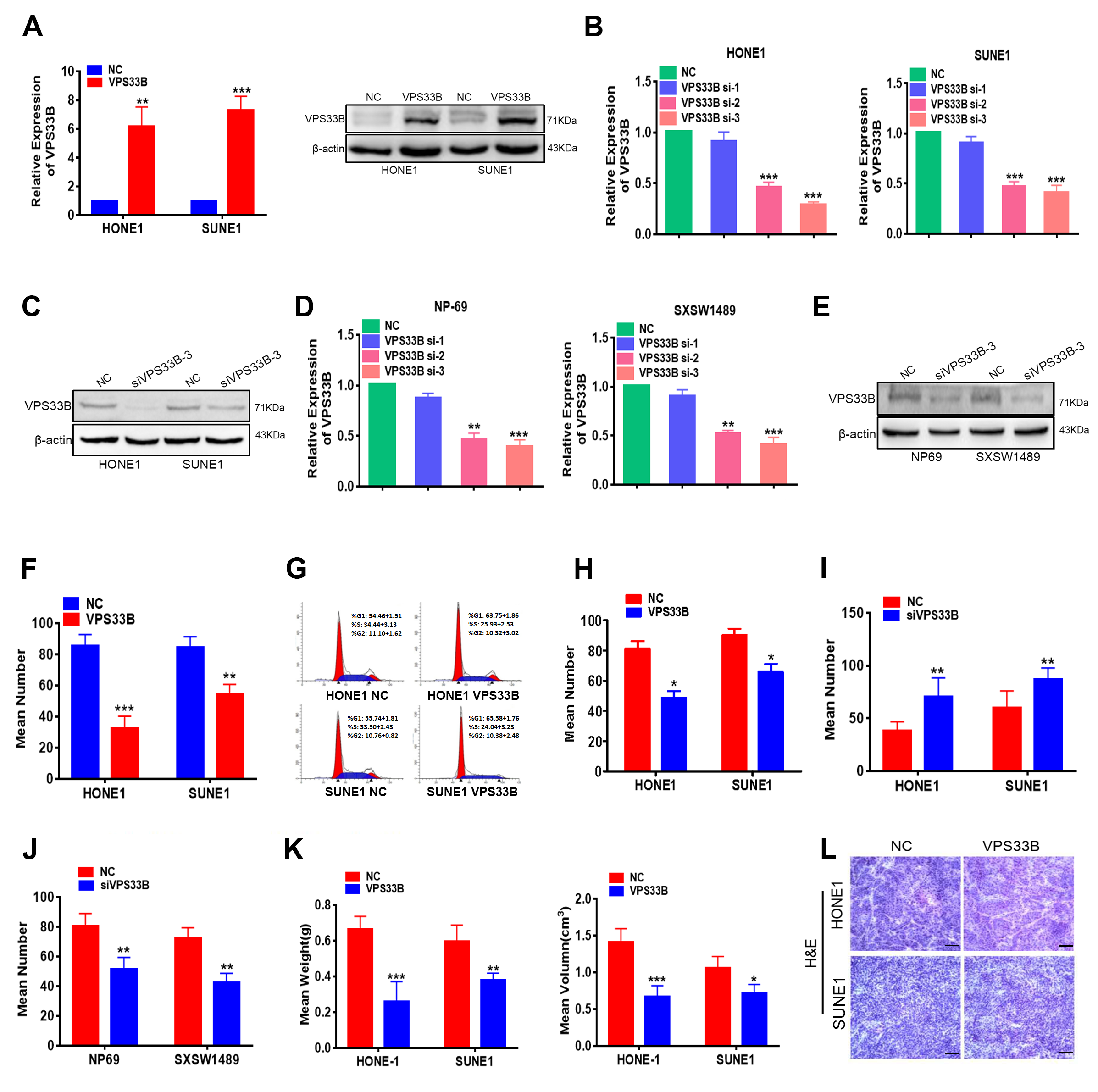

Supplement: Supplementary file 1 — Supplementary Figure 1 [file 41419_2019_1457_MOESM1_ESM.tif]

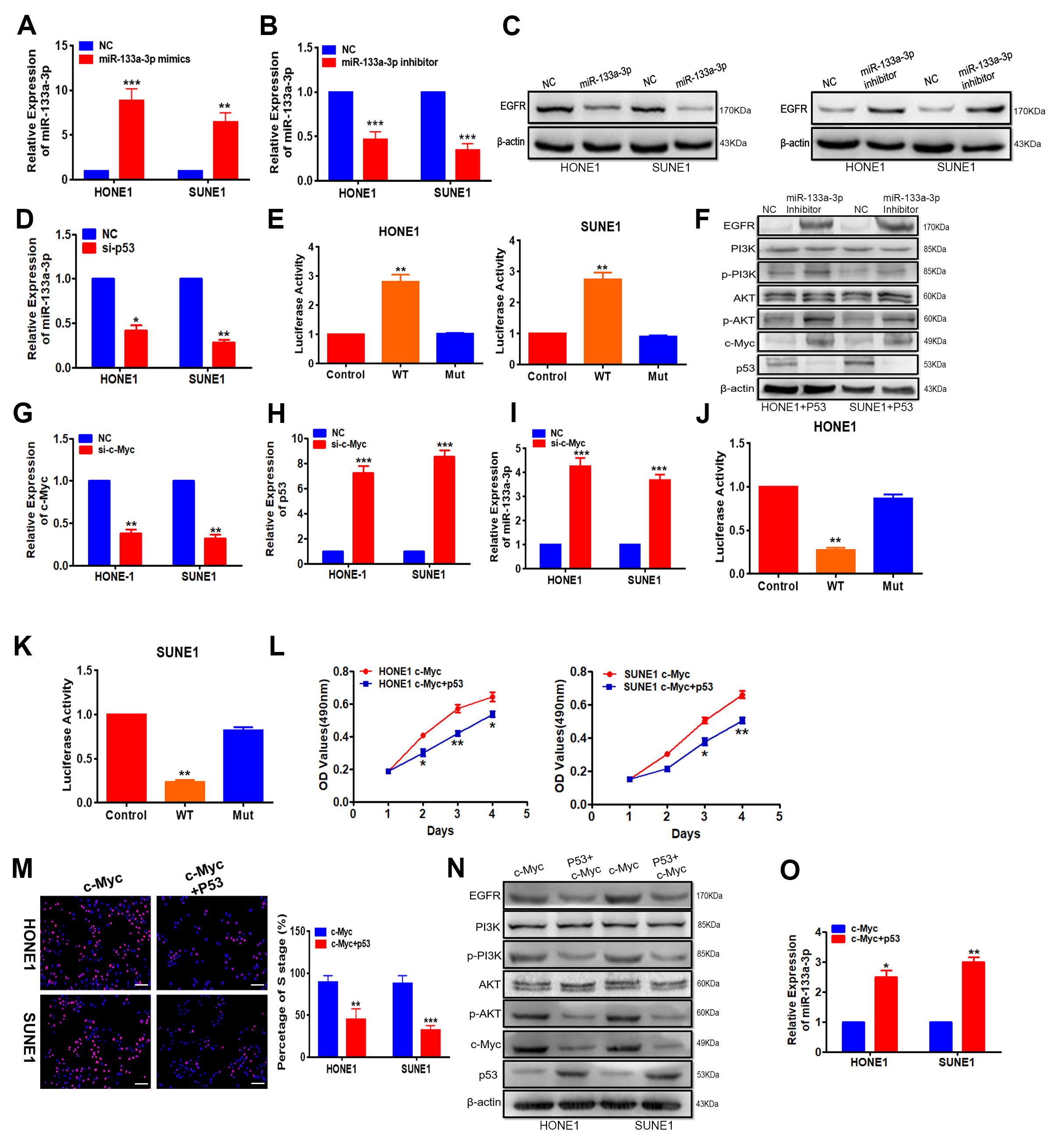

Supplement: Supplementary file 2 — Supplementary Figure 2 [file 41419_2019_1457_MOESM2_ESM.tif]

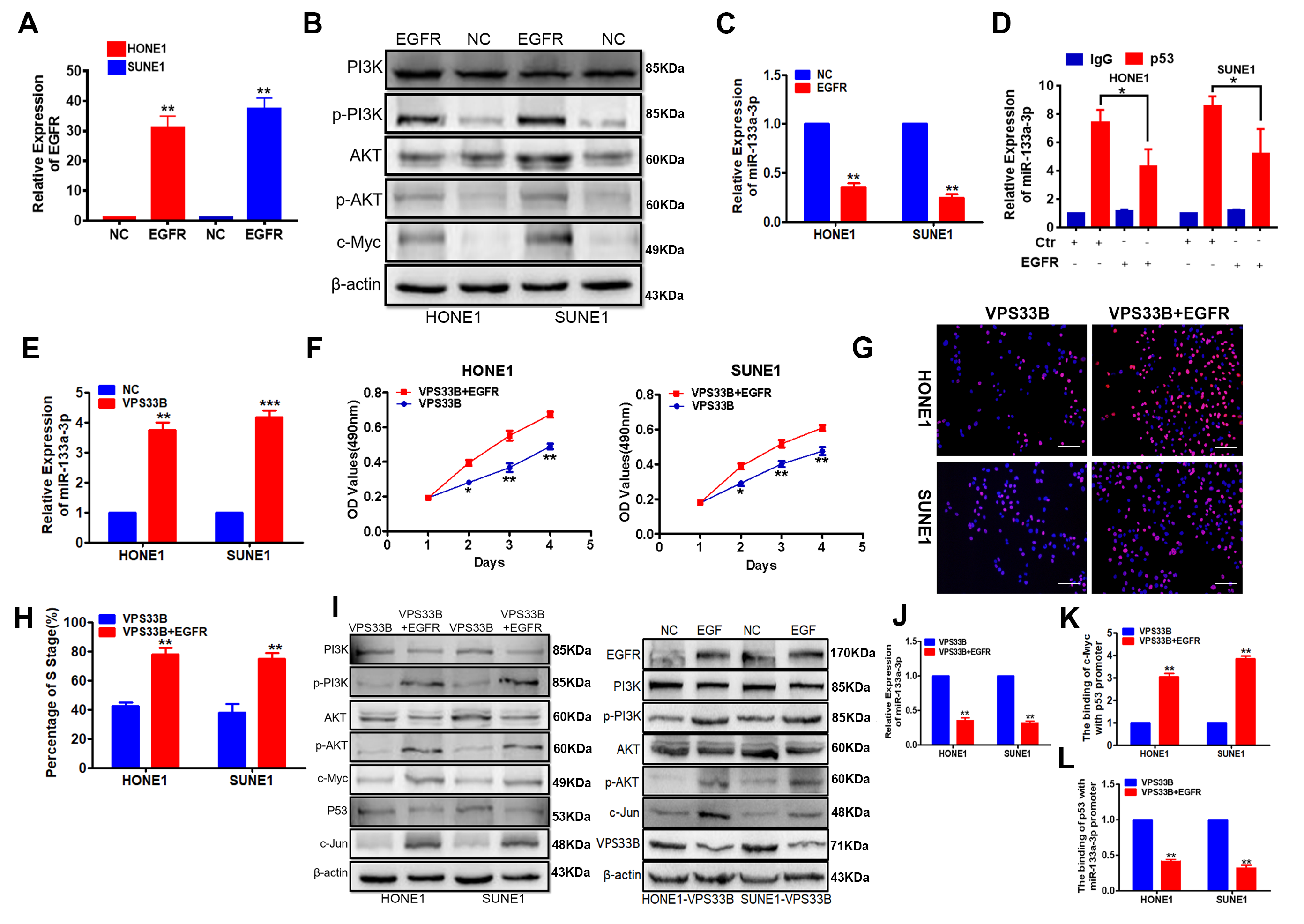

Supplement: Supplementary file 3 — Supplementary Figure 3 [file 41419_2019_1457_MOESM3_ESM.tif]

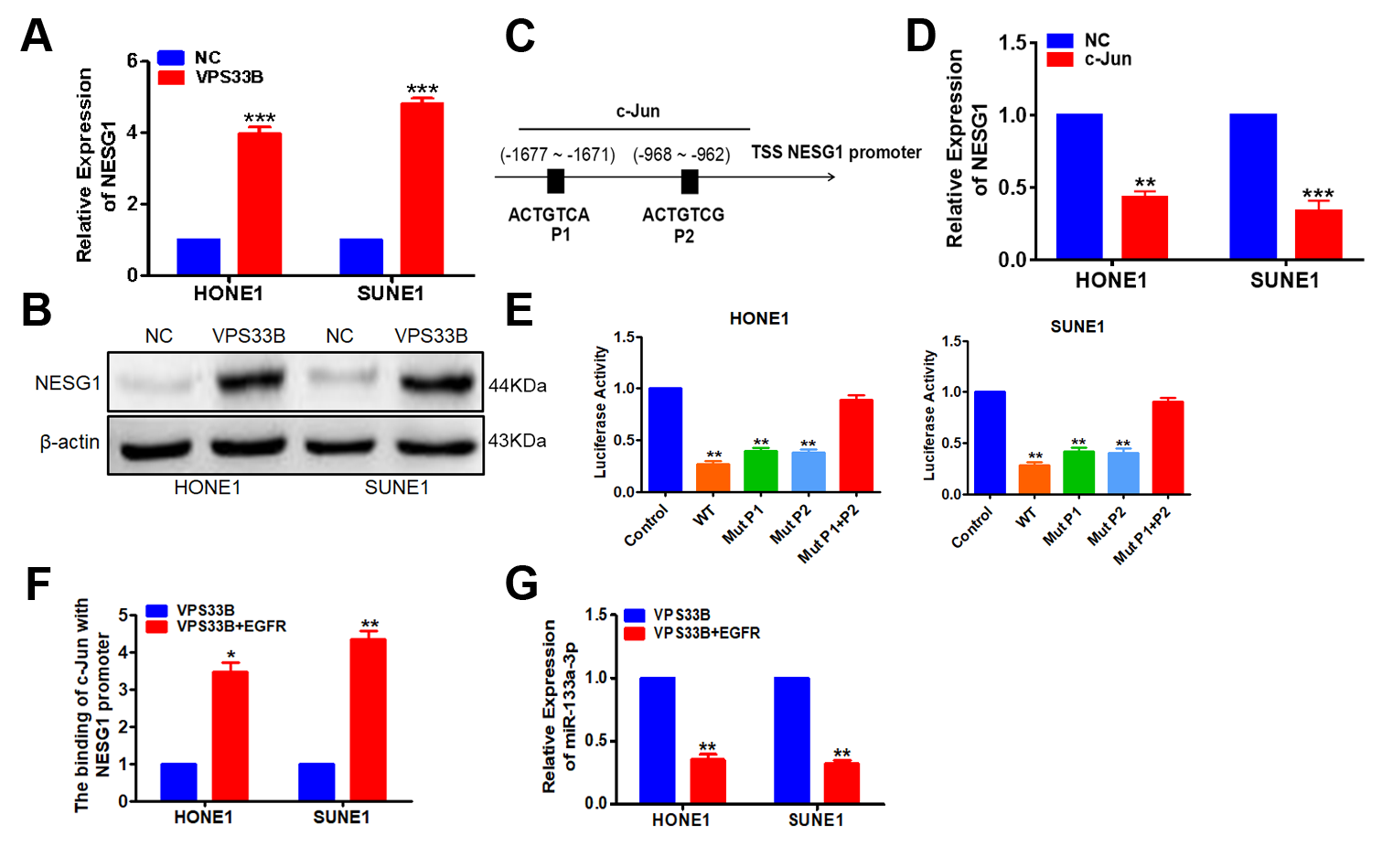

Supplement: Supplementary file 4 — Supplementary Figure 4 [file 41419_2019_1457_MOESM4_ESM.tif]
