# Supplementary material for: VPS33B interacts with NESG1 to modulate EGFR/PI3K/AKT/c-Myc/P53/miR-133a-3p signaling and induce 5-fluorouracil sensitivity in nasopharyngeal carcinoma
Source: Cell Death Dis. 2019 Apr 3;10(4):305. doi: 10.1038/s41419-019-1457-9 (PMC6447525; doi:10.1038/s41419-019-1457-9)
Supplement: Supplementary file 5 — Supplementary Table 1 [file 41419_2019_1457_MOESM5_ESM.doc]

| Primer |  | Sequence |
| --- | --- | --- |
| p53 | Sense | 5’-GAAGAAAAUUUCCGCAAAAdTdT-3’ |
|  | Antisense | 5’-TTTTGCGGAAATTTTCTTCdTdT-3’ |
| c-Myc | Sense | 5’-GGCACAGCUUAAACAGAAA dTdT-3’ |
|  | Antisense | 5’-UUUCUGUUUAAGCUGUGCCdTdT-3’ |
| VPS33B | Sense | 5’-GCGAUACAUUGCCAGUCUUdTdT-3’ |
|  | Antisense | 5’-AAGACUGGCAAUGUAUCGCdTdT-3’ |
| NESG1 | Sense | 5’-GAACGAAGGCAGCAACAAA dTdT-3’ |
|  | Antisense | 5’-UUUGUUGCUGCCUUCGUUCdTdT-3’ |
| miR-133a-3p mimics | Sense | 5’-UUUGGUCCCCUUCAACCAGCUG-3’ |
|  | Antisense | 5’-CUGCUGGUUGUUGGGGUCCUUU-3’ |
| Negative control | Sense | 5’-UUUGUACUACACAAAAGUACUG-3’ |
|  | Antisense | 5’-CUGUUCUUUUGUGUUGUUCUUU-3’ |
| miR-133a-3p inhibitor | | 5’-CUGUGUCCUUUGGUUUUGGCUG-3’ |
| Inhibitor negative control | | 5’-CUGUUCUUUUGUGUUGUUCUUU-3’ |

Supplementary Table 1: The sequences of siRNA, mimics and inhibitor used in this study.
