# Supplementary material for: VPS33B interacts with NESG1 to modulate EGFR/PI3K/AKT/c-Myc/P53/miR-133a-3p signaling and induce 5-fluorouracil sensitivity in nasopharyngeal carcinoma
Source: Cell Death Dis. 2019 Apr 3;10(4):305. doi: 10.1038/s41419-019-1457-9 (PMC6447525; doi:10.1038/s41419-019-1457-9)
Supplement: Supplementary file 6 — Supplementary Table 2 [file 41419_2019_1457_MOESM6_ESM.doc]

| Primers |  | Sequence (5’-3’) |
| --- | --- | --- |
| VPS33B | Forward | ATGAGCCCTTTGGATCGAATTG |
|  | Reverse | ATGCGGGGTCTGACCAAGA |
| NESG1 | Forward | GAGATGGTGTGGAACAACAACA |
|  | Reverse | GGCATGGCACTTAGCATTGAG |
| c-Myc | Forward | GGCTCCTGGCAAAAGGTCA |
|  | Reverse | CTGCGTAGTTGTGCTGATGT |
| P53 | Forward | CAGCACATGACGGAGGTTGT |
|  | Reverse | TCATCCAAATACTCCACACGC |
| EGFR | Forward | AGGCACGAGTAACAAGCTCAC |
|  | Reverse | ATGAGGACATAACCAGCCACC |
| GAPDH | Forward | GCAAATTCCATGGCACCGT |
|  | Reverse | TCGCCCCACTTGATTTTGG |
| miR-133a-3p |  | GCCGAGUUUGGUCCCCUUCA |
| U6 | Forward | CTCGCTTCGGCAGCACA |
|  | Reverse | AACGCTTCACGAATTTGCGT |
| promoter of p53 | Forward | CAAAGGGCAGAGGTACTTTCTT |
|  | Reverse | CTTCTACTGCCACTGCTTCTTT |
| promoter of c-Myc | Forward | ACAGAGAGAGACTCCATCTCAA |
|  | Reverse | CCAACCTTCCCACCACTAAA |
| promoter of NESG1-1 | Forward | GAGGGTTTGGAGAGAGGTTTG |
|  | Reverse | CCACCAGACCAAGGACATTT |
| promoter of NESG1-2 | Forward | ACAGAGAGAGACTCCATCTCAA |
|  | Reverse | ATTGCGGGCCTCAAATCT |
| promoter of VPS33B | Forward | GGCAATAGGTGATGCTCTGTAT |
|  | Reverse | CAGGCTAGCTAACAATACCTCAA |

Supplementary Table 2: The primers used in this study.
