# Supplementary material for: VPS33B interacts with NESG1 to modulate EGFR/PI3K/AKT/c-Myc/P53/miR-133a-3p signaling and induce 5-fluorouracil sensitivity in nasopharyngeal carcinoma
Source: Cell Death Dis. 2019 Apr 3;10(4):305. doi: 10.1038/s41419-019-1457-9 (PMC6447525; doi:10.1038/s41419-019-1457-9)
Supplement: Supplementary file 8 — Supplementary Table 4 [file 41419_2019_1457_MOESM8_ESM.doc]

| Primer name |  | Sequence (5’-3’ ) |
| --- | --- | --- |
| EGFR | Sense | TTCTAGATATGAGCCCTAAAAATCCAGAC |
|  | Antisense | GGGCCGGCCTTCAAAACAATGGAATGGAAGACAA |

Supplementary Table 4: The primer used in Luciferase Report Assay.
