# Supplementary material for: VPS33B interacts with NESG1 to modulate EGFR/PI3K/AKT/c-Myc/P53/miR-133a-3p signaling and induce 5-fluorouracil sensitivity in nasopharyngeal carcinoma
Source: Cell Death Dis. 2019 Apr 3;10(4):305. doi: 10.1038/s41419-019-1457-9 (PMC6447525; doi:10.1038/s41419-019-1457-9)
Supplement: Supplementary file 9 — Supplementary Table 5 [file 41419_2019_1457_MOESM9_ESM.doc]

**Supplementary Table 5: The sequences used in Electrophoretic mobility shift assay.**

| miR-133a-3p | probes | wild type | 5’ CCCATGGCCTCTCCTTGCCCCGATATGGCCAG 3’ |
| --- | --- | --- | --- |
|  | competitors | wild type | 5’ CCCATGGCCTCTCCTTGCCCCGATATGGCCAG 3’ |
|  |  | mutant | 5’ CCCATGGCCTCTCATACATGCGATATGGCCAG 3’ |
| P53 | probes | wild type | 5’CCCACCTGTGCAGCACCTGCACTCCCATGTGCT3’ |
|  | competitors | wild type | 5’CCCACCTGTGCAGCACCTGCACTCCCATGTGCT3’ |
|  |  | mutant | 5’CCACGGACAGCAGGTGGACGACTCGGTACACCT3’ |
| VPS33B | probes | wild type | 5’TCCGCCTCCCTGACTCGAGCGATTCT3’ |
|  | competitors | wild type | 5’TCCGCCTCCCTGACTCGAGCGATTCT3’ |
|  |  | mutant | 5’TCCGCCTCCCACTGAAGAGCGATTCT3’ |
| NESG1 | probes | wild type | 5’ACGCCAGGGCGGGTCAATCCTTCAGAGCCGCGGTTGGGCTGGAGCGTGGAGCTGCAACGGTTGGAGCGACGGGCTCCGGTTCCTAAGCCACCAGCACAAGGCAACTGCCCGCTGGAGGTGGGAGGGGCGGGGCCGGGGGCGGGGCCTGAGCCGAAGAACGCGGGCTAGTGGTTGCCAAGGTAACGCGTCAACACTAGGGCCT3’ |
|  | competitors | wild type | 5’ACGCCAGGGCGGGTCAATCCTTCAGAGCCGCGGTTGGGCTGGAGCGTGGAGCTGCAACGGTTGGAGCGACGGGCTCCGGTTCCTAAGCCACCAGCACAAGGCAACTGCCCGCTGGAGGTGGGAGGGGCGGGGCCGGGGGCGGGGCCTGAGCCGAAGAACGCGGGCTAGTGGTTGCCAAGGTAACGCGTCAACACTAGGGCCT3’ |
|  |  | site 1 mutant | 5’ACGCCAGGGTCAATCGCATCCTTCAGAGCCGCGGTTGGGCTGGAGCGTGGAGCTGCAACGGTTGGAGCGACGGGCTCCGGTTCCTAAGCCACCAGCACAAGGCAACTGCCCGCTGGAGGTGGGAGGGGCGGGGCCGGGGGCGGGGCCTGAGCCGAAGAACGCGGGCTAGTGGTTGCCAAGGTAACGCGTCAACACTAGGGCCT3’ |
|  |  | site 2 mutant | 5’ACGCCAGGGCGGGTCAATCCTTCAGAGCCGCGGTTGGGCTGGAGCGTGGAGCTGCAACGGTTGGAGCGACGGGCTCCGGTTCCTAAGCCACCAGCACAAGGCAACTGCCCGCTGGAGGTGGGAGGGGCGGGGCCGGGGGCGGGGCCTGAGCCGAAGAACGCGGGCTAGTGGTTGCCAAGGTAATATAATGACACTAGGGCCT3’ |
|  |  | all sites mutant | 5’ACGCCAGGGTCAATCGCATCCTTCAGAGCCGCGGTTGGGCTGGAGCGTGGAGCTGCAACGGTTGGAGCGACGGGCTCCGGTTCCTAAGCCACCAGCACAAGGCAACTGCCCGCTGGAGGTGGGAGGGGCGGGGCCGGGGGCGGGGCCTGAGCCGAAGAACGCGGGCTAGTGGTTGCCAAGGTAATATAATGACACTAGGGCCT3’ |
