# Supplementary material for: VPS33B interacts with NESG1 to modulate EGFR/PI3K/AKT/c-Myc/P53/miR-133a-3p signaling and induce 5-fluorouracil sensitivity in nasopharyngeal carcinoma
Source: Cell Death Dis. 2019 Apr 3;10(4):305. doi: 10.1038/s41419-019-1457-9 (PMC6447525; doi:10.1038/s41419-019-1457-9)
Supplement: Supplementary file 10 — supplemental figure legends [file 41419_2019_1457_MOESM10_ESM.docx]

**Supplementary Figure 1. Supplementary figure 1 were related to Figure 1. A.** The mRNA and protein levels of VPS33B were examined by qRT-PCR and western blot after transfecting lentivirus carrying VPS33B cDNA to HONE1 and SUNE1 cells. Student’s t-test, mean ± SD, *P < 0.05, **P < 0.01. **B.** Confirming the effectiveness of silencing VPS33B with specific siRNA in HONE1 and SUNE1 cells by qPCR assay. Student’s t-test, mean ± SD, *P < 0.05, **P < 0.01. **C.** Western blot was used to examine VPS33B protein expression after knocking down VPS33B by selected siRNA in HONE1 and SUNE1 cells. β-actin served as a loading control. **D.** Confirming the effectiveness of silencing VPS33B with specific siRNA in NP69 and SXSW1489 cells by qPCR assay. Student’s t-test, mean ± SD, *P < 0.05, **P < 0.01. **E.** Western blot was used to examine VPS33B protein expression after knocking down VPS33B by selected siRNA in NP69 and SXSW1489 cells. β-actin served as a loading control. **F-H.** Colony formation assay **(F),** Flow Cytometry **(G)** and Edu incorporation assay **(H)** were performed in HONE1 and SUNE1 cells after transfection with lentivirus-mock vector or lentivirus-carrying VPS33B cDNA. **I and J.** Edu incorporation assays were performed after transfecting siRNA against VPS33B in HONE1 and SUNE1 or NP69 and SXSW1489 cells. Student’s t-test, mean ± SD, *P < 0.05, **P < 0.01. **K.** Compared with mock cells, mean tumor weight and volume of HONE1-VPS33B and SUNE1-VPS33B cells were markedly reduced *in vivo*. Student’s t-test, mean ± SD, *P < 0.05. ***P < 0.001. **L.** Representative H&E staining of primary tumor tissues were shown. Scale Bar: 100μm.

**Supplementary Figure 2. A and B.** Confirming the effectiveness of introducing miR-133a-3p mimics or inhibitors to HONE1 and SUNE1 cells respectively by qPCR assays. Student’s t-test, Mean ± SD, *P<0.05, **P<0.01. **C.** Examining the changes in protein of EGFR after introducing miR-133a-3p mimics or inhibitors to HONE1 and SUNE1 cells by western blot. **D.** qPCR assay indicated that miR-133a-3p was upregulated in the p53-overexpressing HONE1 and SUNE1 cells. Student’s t-test, Mean ± SD, *P<0.05, **P<0.01. **E.** Luciferase reporter assay demonstrated the luciferase activities of the wild type, Mut miR-133a-3p promoter in HONE1 and SUNE1 cells transfected with p53 plasmid. Student’s t-test, Mean ± SD, *P<0.05, **P<0.01. **F.** Changes in EGFR, PI3K/AKT/p-PI3K/p-AKT, c-Myc and p53 expression were detected by Western blot analysis in P53-overexpressed HONE1 and SUNE1 cells after transfection of miR-133a-3p mimics. β-actin was used as a loading control. **G.** Confirming the effectiveness of silencing c-Myc in HONE1 and SUNE1 cells with siRNA. Student’s t-test, Mean ± SD, *P<0.05, **P<0.01. **H and I.** p53 and miR-133a-3p were upregulated in the c-Myc-silencing HONE1 and SUNE1 cells by qPCR. Student’s t-test, Mean ± SD, *P<0.05, **P<0.01. **J and K.** Luciferase reporter assay demonstrated the luciferase activities of the wild type, Mut p53 promoter in HONE1 and SUNE1 cells transfected with c-Myc plasmid. Student’s t-test, Mean ± SD, *P<0.05, **P<0.01. **J.** Changes in EGFR, PI3K/AKT/p-PI3K/p-AKT, c-Myc and p53 expression were detected by Western blot analysis in p53-overexpressed HONE1 and SUNE1 cells. β-actin was used as a loading control. **L and M.** MTT **(L)** and Edu incorporation **(M)** assays were performed to demonstrate the impact of miR-133a-3p on the proliferation of A549 and H1975 cells. Student’s t-test, One-way ANOVA, mean ± SD, *P < 0.05, **P < 0.01. Scale Bar: 100μm. **N.** Changes in EGFR, PI3K/AKT/p-PI3K/p-AKT, c-Myc and p53 expression were detected by Western blot analysis in HONE1 and SUNE1 cells after transfection of c-Myc or p53+c-Myc. β-actin was used as a loading control. **O.** Detecting the expression of miR-133a-3p by qPCR assay in HONE1 and SUNE1 cells after transfection of c-Myc or p53+c-Myc. Student’s t-test, Mean ± SD, *P<0.05, **P<0.01.

**Supplementary Figure 3. A.** Confirming the effectiveness of overexpressing EGFR in HONE and SUNE1 cells after transduction of EGFR plasmids. **B.** Changes in EGFR, PI3K/AKT/p-PI3K/p-AKT, c-Myc and p53 expression were detected by Western blot analysis in HONE1 and SUNE1 cells after transfection EGFR plasmids. β-actin was used as a loading control. **C.** miR-133a-3p was confirmed to be downregulated in EGFR-overexpressed HONE1 and SUNE1 cells. Student’s t-test, mean ±s.d, **P < 0.01. **D.** The binding of p53 with miR-133a-3p promoter was identified in EGFR-overexpressed HONE1 and SUNE1 cells after transfecting EGFR plasmids by qPCR assay. Student’s t-test, mean ± SD, **P < 0.01. **E.** miR-133a-3p was demonstrated to be upregulated in VPS33BR-overexpressed HONE1 and SUNE1 cells. Student’s t-test, mean ± SD, **P < 0.01. **F-H.** MTT **(F)** and Edu incorporation **(G-H)** assays were performed to demonstrate the impact of EGFR on the proliferation of VPS33B-overexpressed HONE1 and SUNE1 cells. Student’s t-test, One-way ANOVA, mean ± SD, *P < 0.05, **P < 0.01. Scale Bar: 100μm**. I.** Changes in PI3K/AKT/p-PI3K/p-AKT, c-Myc, p53 and c-Jun expression were detected by Western blot analysis in VPS33B-overexpressed HONE1 and SUNE1 cells after transfection EGFR plasmids. β-actin was used as a loading control. J. Detecting the expression of miR-133a-3p in VPS33B-overexpressed HONE1 and SUNE1 cells transfected with EGFR plasmids by qPCR assay. Student’s t-test, mean ± SD, *P < 0.05, **P < 0.01. **J and K.** The binding of c-Myc with p53 promoter or the binding of p53 with miR-133a-3p promoter were identified in VPS33B-overexpressed HONE1 and SUNE1 cells after transfecting EGFR plasmids by qPCR assay. Student’s t-test, mean ± SD, **P < 0.01.

**Supplementary Figure 4. A and B.** The mRNA level of NESG1 was upregulated in VPS33B-overexpressed HONE1 and SUNE1 cells by qPCR assay. Student’s t-test, mean ± SD, **P < 0.01. ***P < 0.001. **C.** Bioinformatics analysis respectively indicated the putative c-Jun binding site in the promoter regions of NESG1. **D.** The mRNA level of NESG1 was downregulated in c-Jun-overexpressed HONE1 and SUNE1 cells by qPCR assay. Student’s t-test, mean ± SD, *P < 0.05. **E.** Luciferase reporter assay demonstrated the luciferase activities of the wild type, Mut NESG1 promoter in A549 and H1975 cells transfected with c-Jun plasmid. Student’s t-test, Mean ± SD., *P<0.05, **P<0.01. **F.** The binding of c-Jun with NESG1 promoter were identified in VPS33B-overexpressed HONE1 and SUNE1 cells after transfecting EGFR plasmids by qPCR assay. Student’s t-test, mean ± SD, **P < 0.01. **G.** miR-133a-3p was downregulated in VPS33B-overexpressed HONE1 and SUNE1 cells after silencing NESG1 with specific siRNAs by qPCR assay. Student’s t-test, mean ± SD, **P < 0.01.
